# Outcomes of SGLT-2i and GLP-1RA Therapy Among Patients With Type 2 Diabetes and Varying NAFLD Status

**DOI:** 10.1001/jamanetworkopen.2023.49856

**Published:** 2023-12-28

**Authors:** Sungho Bea, Han Eol Jeong, Kristian B. Filion, Oriana HY Yu, Young Min Cho, Bon Hyang Lee, Yoosoo Chang, Christopher D. Byrne, Ju-Young Shin

**Affiliations:** 1 School of Pharmacy, Sungkyunkwan University, Suwon, South Korea; 2 Department of Biohealth Regulatory Science, Sungkyunkwan University, Suwon, South Korea; 3 Departments of Medicine and of Epidemiology, Biostatistics, and Occupational Health, McGill University, Montreal, Quebec, Canada; 4 Centre for Clinical Epidemiology, Lady Davis Institute, Jewish General Hospital, Montreal, Quebec, Canada; 5 Division of Endocrinology and Metabolism, Jewish General Hospital, McGill University, Montreal, Quebec, Canada; 6 Division of Endocrinology, Department of Internal Medicine, Seoul National University Hospital, Seoul, South Korea; 7 Department of Internal Medicine, Seoul National University College of Medicine, Seoul, South Korea; 8 Center for Cohort Studies, Total Healthcare Center, Kangbuk Samsung Hospital Sungkyunkwan University School of Medicine, Seoul, South Korea; 9 Department of Occupational and Environmental Medicine, Kangbuk Samsung Hospital Sungkyunkwan University School of Medicine, Seoul, South Korea; 10 Department of Clinical Research Design & Evaluation, Samsung Advanced Institute for Health Sciences & Technology, Sungkyunkwan University, Seoul, South Korea; 11 Nutrition and Metabolism, Faculty of Medicine, University of Southampton, Southampton, United Kingdom; 12 National Institute for Health and Care Research, Southampton Biomedical Research Centre, University Hospital Southampton, Southampton, United Kingdom

## Abstract

**Importance:**

Nonalcoholic fatty liver disease (NAFLD) is a cardiovascular risk factor, but whether sodium-glucose cotransporter-2 inhibitors (SGLT-2i) and glucagon-like peptide-1 receptor agonists (GLP-1RA) are associated with reduced cardiovascular risk in patients with type 2 diabetes (T2D) and concomitant NAFLD remains uncertain.

**Objective:**

To investigate the outcomes of SGLT-2i and GLP-1RA therapy among patients with T2D varied by the presence or absence of NAFLD.

**Design, Setting, and Participants:**

This retrospective, population-based, nationwide cohort study used an active-comparator new-user design. Two distinct new-user active-comparator cohorts of patients aged 40 years and older who initiated SGLT-2i or GLP-1RA were propensity score matched to patients who initiated dipeptidyl peptidase-4 inhibitors (DPP-4i). The study was conducted in South Korea from January 2013 to December 2020, and data analysis was conducted from October 2022 to March 2023.

**Main Outcomes and Measures:**

The main outcomes were (1) major adverse cardiovascular events (MACE), a composite end point of hospitalization for myocardial infarction, hospitalization for stroke, and cardiovascular death, and (2) hospitalization for heart failure (HHF). Cox proportional hazards models were used to estimate hazard ratios (HRs). The Wald test was applied to assess heterogeneity by NAFLD.

**Results:**

After 1:1 propensity score matching, 140 438 patients were retrieved in the first cohort (SGLT-2i vs DPP-4i; mean [SD] age, 57.5 [10.3] years; 79 633 [56.7%] male) and 34 886 patients were identified in the second cohort (GLP-1RA vs DPP-4i; mean [SD] age, 59.5 [10.5] years; 17 894 [51.3%] male). Compared with DPP-4i, SGLT-2i therapy was associated with a lower risk of MACE (HR, 0.78 [95% CI, 0.71-0.85]) and HHF (HR, 0.62 [95% CI, 0.48-0.81]). GLP-1RA therapy was associated with a decreased risk of MACE (HR, 0.49 [95% CI, 0.39-0.62]) but had statistically nonsignificant findings regarding HHF (HR, 0.64 [95% CI, 0.39-1.07]). Stratified analysis by NAFLD status yielded consistent results for SGLT-2i (MACE with NAFLD: HR, 0.73 [95% CI, 0.62-0.86]; without NAFLD: HR, 0.81 [95% CI, 0.72-0.91]; HHF with NAFLD: HR, 0.76 [95% CI, 0.49-1.17]; without NAFLD: HR, 0.56 [95% CI, 0.40-0.78]) and for GLP-1RA (MACE with NAFLD: HR, 0.49 [95% CI, 0.32-0.77]; without NAFLD: HR, 0.49 [95% CI, 0.37-0.65]; HHF with NAFLD: HR, 0.82 [95% CI, 0.38-1.76]; without NAFLD: HR, 0.54 [95% CI, 0.27-1.06]).

**Conclusions and Relevance:**

In this population-based cohort study, SGLT-2i therapy was associated with a decreased risk of MACE and HHF, while GLP-1RA therapy was associated with a decreased risk of MACE among patients with T2D, irrespective of baseline NAFLD status.

## Key Points

**Question:**

Are sodium-glucose cotransporter-2 inhibitors (SGLT-2i) and glucagon-like peptide-1 receptor agonists (GLP-1RA) associated with reduced cardiovascular risk in patients with type 2 diabetes and concomitant nonalcoholic fatty liver disease (NAFLD)?

**Findings:**

In this population-based cohort study, GLP-1RA and SGLT-2i therapy were associated with reduced risk of major adverse cardiovascular events in patients with T2D and across baseline NAFLD status. Moreover, SGLT-2i therapy was associated with reduced risk of hospitalization for heart failure.

**Meaning:**

These results support the current guidelines that recommend GLP-1RA as the first-line of therapy for patients with T2D and NAFLD and highlight the potential of SGLT-2i as a promising option for cardiovascular disease prevention regardless of NAFLD status.

## Teaser

This cohort study investigates the outcomes of sodium-glucose cotransporter-2 inhibitor (SGLT-2i) and glucagon-like peptide-1 receptor agonist (GLP-1RA) therapy among patients with type 2 diabetes in South Korea, by the presence or absence of nonalcoholic fatty liver disease (NAFLD).

## Introduction

Nonalcoholic fatty liver disease (NAFLD) is a multisystem disease that not only increases risk of liver complications but also increases risk of cardiovascular disease (CVD).^1,2^ Notably, the prevalence of NAFLD is much higher among patients with type 2 diabetes (T2D). These chronic diseases share multiple risk factors, such as abdominal obesity and insulin resistance.^3,4,5^ Furthermore, the bidirectional relationship between NAFLD and T2D interferes with glycemic control, subsequently increasing the risk of extrahepatic complications, including cardiovascular disease.^5,6,7,8^ Despite NAFLD being an emerging threat to global public health, affecting approximately 25% of the general population worldwide, there is a lack of evidence regarding the prevention of macrovascular complications in individuals who have both NAFLD and T2D.^9^

Glucose-lowering medications, such as sodium-glucose cotransporter-2 inhibitors (SGLT-2i) and glucagon-like peptide-1 receptor agonists (GLP-1RA), have shown cardiorenal benefits beyond blood glucose control.^10,11,12,13,14,15,16,17^ Moreover, several studies have demonstrated the potential hepatic benefits of these drugs across the spectrum of liver disease including liver steatosis, liver fibrosis, and hepatocellular carcinoma.^18,19,20,21,22,23^ SGLT-2is may decrease hepatic steatosis, inflammation, or fibrosis through glucagon signaling pathways or insulin resistance (hyperinsulinemia),^24,25,26^ whereas GLP-1RAs reduce insulin resistance, weight loss, and gut-liver axis modulation.^27^ Despite the established biological mechanisms of these novel glucose-lowering medication classes, their clinical effectiveness among patients with both NAFLD and T2D remains unknown.

While there is a higher CVD risk among patients with NAFLD and T2D, no studies have considered NAFLD as a key variable in evaluating the effectiveness of glucose-lowering medications.^28^ Given the increasing burden of liver-related comorbidities in patients with T2D, our objective was to investigate whether NAFLD modifies the association of SGLT-2i and GLP-1RA with reduced risk of CVD by stratifying patients with T2D based on their baseline NAFLD status.

## Methods

### Target Trial Emulation

This cohort study aimed to emulate a hypothetical target trial by implementing a target trial design, with the objective of replicating key features of a randomized clinical trial using observational data^29^ (eTable 1 in Supplement 1). By using this design, we sought to enhance the accuracy of causal effect estimates in our study. This study underwent evaluation by the institutional review board at Sungkyunkwan University and followed the Strengthening the Reporting of Observational Studies in Epidemiology (STROBE) guideline. The requirement for informed consent was waived, as this study used anonymized administrative data.

### Data Source

We conducted a nationwide, population-based cohort study using health insurance claims data from the National Health Insurance Service (NHIS) database of South Korea. South Korea has universal health insurance coverage provided by a single-payer system that covers approximately 50 million residents.^30^ The NHIS database contains sociodemographic information, medical diagnoses, inpatient and outpatient prescriptions, and biennial health examination results.^30^ Diagnoses were documented using the *International Statistical Classification of Diseases and Related Health Problems, Tenth Revision *(*ICD-10*) coding system. Drugs are documented using domestic National Health Insurance coding systems, including information on the active ingredient, dose, route of administration, prescription date, and number of days supplied. Health examinations included anthropometric data, laboratory measurements, and self-reported questionnaires.

### Study Population

We conducted an active comparator, new-user cohort study by assembling 2 independent cohorts. The first cohort comprised of new users of SGLT-2i and DPP-4i between September 1, 2014 (the first date of insurance coverage for SGLT-2i in South Korea), and December 31, 2020. The second cohort comprised new users of GLP-1RA and DPP-4i between January 1, 2013 (the earliest date of the NHIS database for eligibility criteria), and December 31, 2020. We identified each cohort independently, ensuring that inclusion in one cohort was not dependent on inclusion in the other.

Cohort entry was defined by the date of first prescription of either the drug of interest (SGLT-2i or GLP-1RA) or DPP-4i within each cohort. We assumed that patients had T2D, as these medications are reimbursed exclusively for T2D. All patients were required to have undergone at least 1 health examination in the 3 years before cohort entry for calculating fatty liver index (FLI). We compared differences in baseline characteristics between patients with T2D who had a health examination record vs patients with T2D to assess that patients with a health screening record could represent the overall population of patients with T2D (eTable 2 in Supplement 1). In addition, the duration between the latest health examination and cohort entry was measured to ensure the validity of the health examination records (eTable 3 in Supplement 1). Subsequently, we excluded patients younger than 40 years at the time of cohort entry. We further excluded those prescribed both the drug of interest and a DPP-4i on the date of cohort entry and those who received any drug of interest (in the pairwise comparison) or DPP-4i in the year before cohort entry. We also excluded patients with a record of end-stage kidney disease or receiving dialysis in the year before cohort entry. Finally, we excluded patients with a history of liver-related diseases (ie, at least 1 inpatient or outpatient diagnostic code for alcoholic liver disease, viral hepatitis, or autoimmune liver disease) in the year before cohort entry.^31^

Among all patients prescribed the drug of interest and DPP-4i for the management of T2D, we stratified patients based on the presence or absence of NAFLD. Based on the most recent health examination results, we used FLI as a surrogate indicator of NAFLD, which is extensively validated as an alternative metric to liver image screening in large population-based epidemiologic studies.^32^ Moreover, the FLI has been previously validated in the Korean population, with a PPV of 89% (eAppendix 1 in Supplement 1).^33^ We used an FLI cutoff value of 60 to define NAFLD, in line with previous studies.^32,33,34^ Notably, patients with diagnostic codes for alcoholic liver disease, viral hepatitis, autoimmune liver disease, or other secondary steatogenic conditions were excluded from our study. This exclusion aimed to rule out patients with any other liver disease aside from NAFLD. Therefore, the patients with an FLI of 60 or greater were considered to have FLI-diagnosed NAFLD (referred to as NAFLD hereafter).

### Exposure

We applied an as-treated approach to define exposure, in which patients were followed up from the day after the initiation of cohort entry–defining drugs (SGLT-2i, GLP-1RA, or DPP-4i) until the occurrence of a study outcome (defined later), censoring due to discontinuation (or switching) of the cohort entry–defining drug, death, or end of the study period (December 31, 2020), whichever came first. Treatment discontinuation was defined as a gap of more than 60 days between successive prescriptions.

DPP-4is were chosen as the comparator drug of interest due to their similarity to SGLT-2is and GLP-1RAs as second- or third-line antidiabetic therapies for the management of T2D. The selection of DPP-4i as the comparator aimed to mitigate potential biases arising from confounding factors, such as indication bias or variations in disease severity among patients with T2D.^35^ Furthermore, a randomized trial among patients with NAFLD or nonalcoholic steatohepatitis (NASH) also showed that DPP-4i has either no or limited effects on hepatic outcomes; DPP-4i also has neutral effects on weight loss and anti-inflammation, which are important factors in the management of NAFLD and T2D.^36^

### Outcomes

We evaluated the coprimary effectiveness outcomes of (1) major adverse cardiovascular events (MACE), a composite end point of hospitalization for myocardial infarction, hospitalization for stroke, and cardiovascular death, and (2) hospitalization for heart failure (HHF). Our secondary outcomes were the individual end points of MACE and all-cause mortality. All outcomes were defined using a diagnosis in the primary (most accountable) position of the hospitalization record.^37^ The positive predictive value of these claims databased outcomes range from 82.1% to 92.0% (eTable 4 in Supplement 1).

### Potential Confounders

We assessed the baseline demographic covariates of age and sex at cohort entry. Diabetes-related covariates were also measured, including diabetic complications, class of concomitant antidiabetic drug use, and level of antidiabetic treatment, as a proxy variable for diabetes severity, which was categorized into 3 mutually exclusive groups based on the class and number of different antidiabetic drugs in the preceding year of cohort entry: (1) patients who had not received any antidiabetic treatment or who had received only 1 antidiabetic agent; (2) patients who had received 2 or more different classes of antidiabetic drugs without insulin; and (3) patients who had received insulin either unaccompanied or in combination with other antidiabetic drugs. Moreover, we assessed various comorbidities, use of comedications, and measures of general health care utilization, all measured within a year before cohort entry (eTable 5 in Supplement 1).

### Statistical Analysis

Baseline characteristics of the initiators of the drug of interest and DPP-4i were compared using descriptive statistics. To adjust for potential confounding factors, we applied 1:1 propensity score (PS) matching within each stratum based on the presence of NAFLD (ie, with vs without NAFLD). The PS is the likelihood of receiving the drug of interest vs DPP-4i, estimated through a multivariable logistic regression model, with all covariates measured at baseline included as independent variables. The greedy nearest-neighbor algorithm was applied to the PS with a caliper of 0.05 on the log (PS) scale. Potential imbalances in baseline covariates between each exposure group were evaluated using absolute values of standardized mean differences (SMDs), with values less than 0.1 indicating an acceptable balance.^38^

For all effectiveness outcomes, we estimated absolute risk differences (RDs) along with 95% CIs between the treatment groups. These estimations were based on the incidence rate calculated using the Poisson distribution per 1000 person-years. Cox proportional regression hazard models were used to estimate hazard ratios (HRs) with 95% CIs for the study outcomes associated with SGLT-2i or GLP-1RA vs DPP-4i users. The Schoenfeld residuals were measured to test the proportional hazard assumption. The cumulative incidence curves of the coprimary effectiveness outcomes were plotted to visualize the time to each outcome. The Wald test for interaction was applied to assess whether outcomes changes based on NAFLD status across the 2 subcohorts.

We conducted 7 sensitivity analyses to evaluate the robustness of our main findings. First, we applied an intention-to-treat approach to consider potential informative censoring due to drug discontinuation or switching. We followed up patients from the initiation of the study drugs until the occurrence of an outcome or censoring due to death, 365 days of follow-up, or the end of the study period. Second, the main analysis was repeated using the hepatic steatosis index, an alternative NAFLD definition, to minimize any potential misclassification of patients with NAFLD that would have arisen with the FLI (eAppendix 1 in Supplement 1).^39^ Third, we restricted our inclusion criteria to patients who underwent a health examination within 1 year before cohort entry to improve the FLI measurement accuracy. Fourth, a competing risk analysis was conducted that treated death as a competing event by using the Fine and Gray model.^40^ Fifth, a PS fine stratification weighting method was applied to explore the average treatment effect of the whole population (ATE), in contrast to the main analysis that estimated the average treatment effect among the treated population (ATT).^41^ Finally, the grace period used to define treatment discontinuation varied between 45 and 90 days to prevent the potential misclassification of exposure.

The statistical analyses were performed utilizing SAS software version 9.4 (SAS Institute), and a 2-sided *P* ≤ .05 was deemed statistically significant. Data analysis was performed from October 2022 to March 2023.

## Results

### SGLT-2i vs DPP-4i

Before matching, the overall cohort of patients treated with SGLT-2i vs DPP-4i included 214 453 patients with NAFLD and 466 674 patients without NAFLD (eFigure 1 in Supplement 1). Overall, mean (SD) age was 61.6 (10.7) years, and 57.2% of patients were male (eTable 6 in Supplement 1). After 1:1 PS matching, mean age was 57.5 (SD 10.3) years and 389 849 patients (56.7%) were male among the overall population (Table 1). While new users of SGLT-2i were younger, more likely to have dyslipidemia, and less likely to have received antidiabetic medications (eg, metformin and sulfonylurea) before matching, balance was achieved for all baseline covariates included in the PS model between the 2 exposure groups after matching (Table 1; eTable 6 in Supplement 1). Follow-up durations varied according to each outcome; mean (SD) follow-up durations overall were 1.6 (1.5) years for MACE (SGLT-2i, 1.4 [1.4] years; DPP-4i, 1.7 [1.6] years) and 1.6 (1.5) years for HHF (SGLT-2i, 1.5 [1.4] years; DPP-4i, 1.7 [1.6] years).

**Table 1.** Baseline Characteristics of 1:1 Propensity Score–Matched Patients Initiating SGLT-2is vs DPP-4is Overall and Across NAFLD Status

Characteristic	Patients with NAFLD			Patients without NAFLD			Overall population		
	SGLT-2i, No. (%)	DPP-4i, No. (%)	SMD	SGLT-2i, No. (%)	DPP-4i, No. (%)	SMD	SGLT-2i, No. (%)	DPP-4i, No. (%)	SMD
Patients, No.	30 040	30 040	NA	40 179	40 179	NA	70 219	70 219	NA
Fatty liver index, mean (SD)	79.3 (11.1)	78.2 (10.9)	0.001	33.4 (15.8)	31.2 (16.1)	0.021	53.0 (26.7)	51.3 (27.2)	0.031
Age, mean (SD), y	54.7 (9.6)	54.5 (9.9)	0.023	59.8 (10.1)	59.6 (10.4)	0.016	57.6 (10.2)	57.4 (10.5)	0.018
Sex									
Male	20 096 (66.9)	20 337 (67.7)	0.017	19 593 (48.8)	19 607 (48.8)	0.001	39 689 (56.5)	39 944 (56.9)	0.007
Female	9944 (33.1)	9703 (32.3)		20 586 (51.2)	20 572 (51.2)		30 530 (43.5)	30 275 (43.1)	
Calendar year									
2014	732 (2.4)	698 (2.3)	0.004	1323 (3.3)	1367 (3.4)	0.003	2055 (2.9)	2065 (2.9)	0.001
2015	2645 (8.8)	2609 (8.7)		4060 (10.1)	4073 (10.1)		6705 (9.5)	6682 (9.5)	
2016	3816 (12.7)	3779 (12.6)		5618 (14.0)	5506 (13.7)		9434 (13.4)	9285 (13.2)	
2017	5086 (16.9)	5131 (17.1)		7135 (17.8)	7071 (17.6)		12 221 (17.4)	12 202 (17.4)	
2018	5020 (16.7)	5032 (16.8)		6292 (15.7)	6271 (15.6)		11 312 (16.1)	11 303 (16.1)	
2019	6365 (21.2)	6431 (21.4)		8098 (20.2)	8108 (20.2)		14 463 (20.6)	14 539 (20.7)	
2020	6376 (21.2)	6360 (21.2)		7653 (19.0)	7783 (19.4)		14 029 (20.0)	14 143 (20.1)	
Health care use^a^									
Inpatient hospitalizations, No.									
0	24 491 (81.5)	24 644 (82.0)	0.085	31 828 (79.2)	32 018 (79.7)	0.026	56 319 (80.2)	56 662 (80.7)	0.026
1-2	5049 (16.8)	4908 (16.3)		7521 (18.7)	7321 (18.2)		12 570 (17.9)	12 229 (17.4)	
≥3	500 (1.7)	488 (1.6)		830 (2.1)	840 (2.1)		1330 (1.9)	1328 (1.9)	
Physician visits, No.									
0-2	1659 (5.5)	1801 (6.0)	0.082	1571 (3.9)	1639 (4.1)	0.041	3230 (4.6)	3440 (4.9)	0.067
3-5	2749 (9.2)	2853 (9.5)		2568 (6.4)	2625 (6.5)		5317 (7.6)	5478 (7.8)	
≥6	25 632 (85.3)	25 386 (84.5)		36 040 (89.7)	35 915 (89.4)		61 672 (87.8)	61 301 (87.3)	
Comorbidities^a^									
Dyslipidemia	12 859 (42.8)	12 596 (41.9)	0.018	17 167 (42.7)	16 885 (42.0)	0.014	30 026 (42.8)	29 481 (42.0)	0.016
Hypertension	16 367 (54.5)	16 037 (53.4)	0.022	18 737 (46.6)	18 102 (45.1)	0.032	35 104 (50.0)	34 139 (48.6)	0.027
Atrial fibrillation	402 (1.3)	389 (1.3)	0.004	710 (1.8)	664 (1.7)	0.009	1112 (1.6)	1053 (1.5)	0.007
Heart failure	69 (0.2)	71 (0.2)	-.001	69 (0.2)	58 (0.1)	0.007	138 (0.2)	129 (0.2)	0.003
Liver cirrhosis	160 (0.5)	139 (0.5)	0.010	280 (0.7)	288 (0.7)	-.002	440 (0.6)	427 (0.6)	0.002
Chronic kidney disease	75 (0.2)	67 (0.2)	0.005	254 (0.6)	248 (0.6)	0.002	329 (0.5)	315 (0.4)	0.003
Dementia	1188 (4.0)	1159 (3.9)	0.005	1805 (4.5)	1725 (4.3)	0.010	2993 (4.3)	2884 (4.1)	0.008
Depression	703 (2.3)	680 (2.3)	0.005	1225 (3.0)	1190 (3.0)	0.005	1928 (2.7)	1870 (2.7)	0.005
Hypothyroidism	207 (0.7)	199 (0.7)	0.003	442 (1.1)	460 (1.1)	-.004	649 (0.9)	659 (0.9)	-.001
Hyperthyroidism	564 (1.9)	533 (1.8)	0.008	715 (1.8)	719 (1.8)	-.001	1279 (1.8)	1252 (1.8)	0.003
Gallbladder disease	1341 (4.5)	1308 (4.4)	0.005	2241 (5.6)	2125 (5.3)	0.013	3582 (5.1)	3433 (4.9)	0.010
COPD	1675 (5.6)	1619 (5.4)	0.008	2802 (7.0)	2747 (6.8)	0.005	4477 (6.4)	4366 (6.2)	0.007
Comedication^a^									
Acetaminophen	17 481 (58.2)	17 287 (57.5)	0.013	24 836 (61.8)	24 862 (61.9)	-.001	42 317 (60.3)	42 149 (60.0)	0.005
RAS inhibitors	16 247 (54.1)	15 806 (52.6)	0.029	18 078 (45.0)	17 568 (43.7)	0.026	34 325 (48.9)	33 374 (47.5)	0.027
CCB	12 701 (42.3)	12 475 (41.5)	0.015	13 808 (34.4)	13 478 (33.5)	0.017	26 509 (37.8)	25 953 (37.0)	0.016
β-Blockers	5307 (17.7)	5144 (17.1)	0.014	6654 (16.6)	6438 (16.0)	0.015	11 961 (17.0)	11 582 (16.5)	0.014
Diuretics	7490 (24.9)	7314 (24.3)	0.014	8538 (21.2)	8136 (20.2)	0.025	16 028 (22.8)	15 450 (22.0)	0.020
Systemic antibiotics	19 565 (65.1)	19 406 (64.6)	0.011	26 930 (67.0)	27 122 (67.5)	-.010	46 495 (66.2)	46 528 (66.3)	-.001
Oral anticoagulants	414 (1.4)	410 (1.4)	0.001	778 (1.9)	720 (1.8)	0.011	1192 (1.7)	1130 (1.6)	0.007
Oral antiplatelets	7360 (24.5)	7120 (23.7)	0.019	12 235 (30.5)	11 891 (29.6)	0.019	19 595 (27.9)	19 011 (27.1)	0.019
NSAIDs	18 224 (60.7)	17 983 (59.9)	0.016	25 687 (63.9)	25 756 (64.1)	-.004	43 911 (62.5)	43 739 (62.3)	0.005
Opioids	2798 (9.3)	2724 (9.1)	0.009	4048 (10.1)	3910 (9.7)	0.011	6846 (9.7)	6634 (9.4)	0.010
Systemic corticosteroids	14 795 (49.3)	14 690 (48.9)	0.007	21 269 (52.9)	21 097 (52.5)	0.009	36 064 (51.4)	35 787 (51.0)	0.008
Statins	15 546 (51.8)	15 058 (50.1)	0.033	22 566 (56.2)	22 101 (55.0)	0.023	38 112 (54.3)	37 159 (52.9)	0.027
Other lipid-lowering agents	5903 (19.7)	5742 (19.1)	0.014	6298 (15.7)	6096 (15.2)	0.014	12 201 (17.4)	11 838 (16.9)	0.014
Vitamin E	2279 (7.6)	22,10 (7.4)	0.009	2200 (5.5)	2166 (5.4)	0.004	4479 (6.4)	4376 (6.2)	0.006
Nitrates	1492 (5.0)	1440 (4.8)	0.008	2739 (6.8)	2612 (6.5)	0.013	4231 (6.0)	4052 (5.8)	0.011
Antidiabetic drugs use^a^									
Insulin	1965 (6.5)	1856 (6.2)	0.015	3809 (9.5)	3789 (9.4)	0.002	5774 (8.2)	5645 (8.0)	0.007
α-Glucosidase inhibitors	545 (1.8)	556 (1.9)	-.003	1465 (3.6)	1428 (3.6)	0.005	2010 (2.9)	1984 (2.8)	0.002
GLP-1RA	96 (0.3)	81 (0.3)	0.009	104 (0.3)	95 (0.2)	0.005	200 (0.3)	176 (0.3)	0.007
Meglitinides	80 (0.3)	76 (0.3)	0.003	241 (0.6)	224 (0.6)	0.006	321 (0.5)	300 (0.4)	0.005
Metformin	15 063 (50.1)	14 737 (49.1)	0.022	23 673 (58.9)	23 391 (58.2)	0.014	38 736 (55.2)	38 128 (54.3)	0.017
Sulfonylureas	6086 (20.3)	5885 (19.6)	0.017	10 762 (26.8)	10 672 (26.6)	0.005	16 848 (24.0)	16 557 (23.6)	0.010
Thiazolidinediones	1689 (5.6)	1587 (5.3)	0.015	3102 (7.7)	3019 (7.5)	0.008	4791 (6.8)	4606 (6.6)	0.011
Level of antidiabetic treatments^b^									
1	22 601 (75.2)	22 853 (76.1)	0.041	26 712 (66.5)	26 863 (66.9)	0.035	49 313 (70.2)	49 716 (70.8)	0.025
2	5474 (18.2)	5331 (17.7)		9658 (24.0)	9527 (23.7)		15 132 (21.5)	14 858 (21.2)	
3	1965 (6.5)	1856 (6.2)		3809 (9.5)	3789 (9.4)		5774 (8.2)	5645 (8.0)	
Diabetic complications^a^									
Retinopathy	790 (2.6)	761 (2.5)	0.006	1279 (3.2)	1250 (3.1)	0.004	2069 (2.9)	2011 (2.9)	0.005
Neuropathy	2413 (8.0)	2362 (7.9)	0.006	4545 (11.3)	4398 (10.9)	0.012	6958 (9.9)	6760 (9.6)	0.009
Nephropathy	3005 (10)	2832 (9.4)	0.019	6358 (15.8)	6200 (15.4)	0.011	9363 (13.3)	9032 (12.9)	0.014
CCI groups^a^									
0	9593 (31.9)	9824 (32.7)	0.027	9919 (24.7)	10 024 (24.9)	0.024	19 512 (27.8)	19 848 (28.3)	0.024
1-2	13 606 (45.3)	13 605 (45.3)		18 207 (45.3)	18 404 (45.8)		31 813 (45.3)	32 009 (45.6)	
≥3	6841 (22.8)	6611 (22.0)		12 053 (30.0)	11 751 (29.2)		18 894 (26.9)	18 362 (26.1)	
Health examination data, mean (SD)									
BMI	29.7 (15.8)	28.9 (3.6)	0.130	25.1 (2.7)	24.4 (2.7)	0.005	27.1 (3.9)	26.3 (3.8)	0.005
WC, cm	96.2 (9.9)	94.4 (8.1)	0.005	84.3 (6.9)	83.0 (7.1)	0.015	89.4 (10.2)	87.9 (9.4)	0.030
Total cholesterol, mg/dL	204.0 (52.0)	207.0 (54.4)	0.015	185.9 (46.5)	187.8 (46.4)	0.005	193.6 (49.7)	196.0 (50.9)	0.010
HDL cholesterol, mg/dL	48.3 (13.8)	48.4 (22.0)	0.090	52.5 (14.5)	52.4 (14.4)	0.003	50.7 (14.4)	50.7 (18.1)	0.035
LDL cholesterol, mg/dL	110.4 (47.0)	110.9 (47.9)	0.010	106.9 (42.9)	108.2 (42.6)	0.008	108.4 (44.7)	109.4 (44.9)	0.010
TG, mg/dL	255.2 (205.7)	270.3 (216.5)	0.055	133.8 (70.3)	137.7 (74.7)	0.055	185.7 (156.6)	194.4 (166.0)	0.132
Serum creatinine, mg/dL	0.9 (0.4)	0.9 (0.6)	0.005	0.9 (0.7)	0.9 (0.5)	0.005	0.9 (0.6)	0.9 (0.5)	0.005
AST, IU/L	39.3 (32.1)	39.1 (28.6)	0.085	27.1 (15.8)	26.7 (15.5)	0.005	32.3 (24.9)	32.0 (22.9)	0.005
ALT, IU/L	48.0 (39.3)	47.2 (36.0)	0.013	28.9 (20.2)	28.2 (20.0)	0.005	37.1 (30.3)	36.3 (29.4)	0.005
GGT, U/L	83.8 (87.6)	93.1 (100.9)	0.118	33.9 (28.3)	35.2 (32.3)	0.005	55.2 (65.9)	59.9 (76.0)	0.003
GFR, mL/min/1.73 m^2^	90.6 (27.2)	90.4 (28.7)	0.045	88.8 (27.2)	88.7 (28.8)	0.003	89.6 (27.2)	89.4 (28.8)	0.008
FBG, mg/dL	152.0 (51.6)	157.3 (55.4)	0.058	141.1 (48.9)	146.8 (54.0)	0.128	145.7 (50.4)	151.3 (54.8)	0.035

Abbreviations: ALT, serum alanine transaminase; AST, serum aspartate transaminase; BMI, body mass index (calculated as weight in kilograms divided by height in meters squared); CCB, calcium channel blocker; CCI, Charlson Comorbidity Index; COPD, chronic obstructive pulmonary disease; DPP-4i, dipeptidyl peptidase 4 inhibitor; FBG, fasting blood glucose level; GFR, glomerular filtration rate; GGT, γ-glutamyltransferase; GLP-1RA, glucagon-like peptide-1 receptor agonist; HDL, high-density lipoprotein; LDL, low-density lipoprotein; NA, not applicable; NAFLD, nonalcoholic fatty liver disease; NSAID, nonsteroidal anti-inflammatory drug; RAS, renin-angiotensin system; SMD, standardized mean difference; SGLT-2i, sodium glucose cotransporter-2 inhibitor; TG, triglyceride; WC, waist circumference.

SI conversion factors: To convert ALT, AST, and GGT to microkatals per liter, multiply by 0.0167; FBG to millimoles per liter, multiply by 0.0555; HDL, LDL, and total cholesterol to millimoles per liter, multiply by 0.0259; serum creatinine to micromoles per liter, multiply by 88.4; and TG to millimoles per liter, multiply by 0.0113.

^a^ Assessed in the years before study cohort entry.

^b^ Use of antidiabetic drugs previous 365 days before the entry date of cohort: level 1, patients who had not received any antidiabetic drugs or received only 1 antidiabetic; level 2, patients who had received at least 2 different classes of antidiabetic drugs without insulin; level 3, patients who had received at least 1 insulin prescription either unaccompanied or in combination with other antidiabetics.

**Figure 1.**
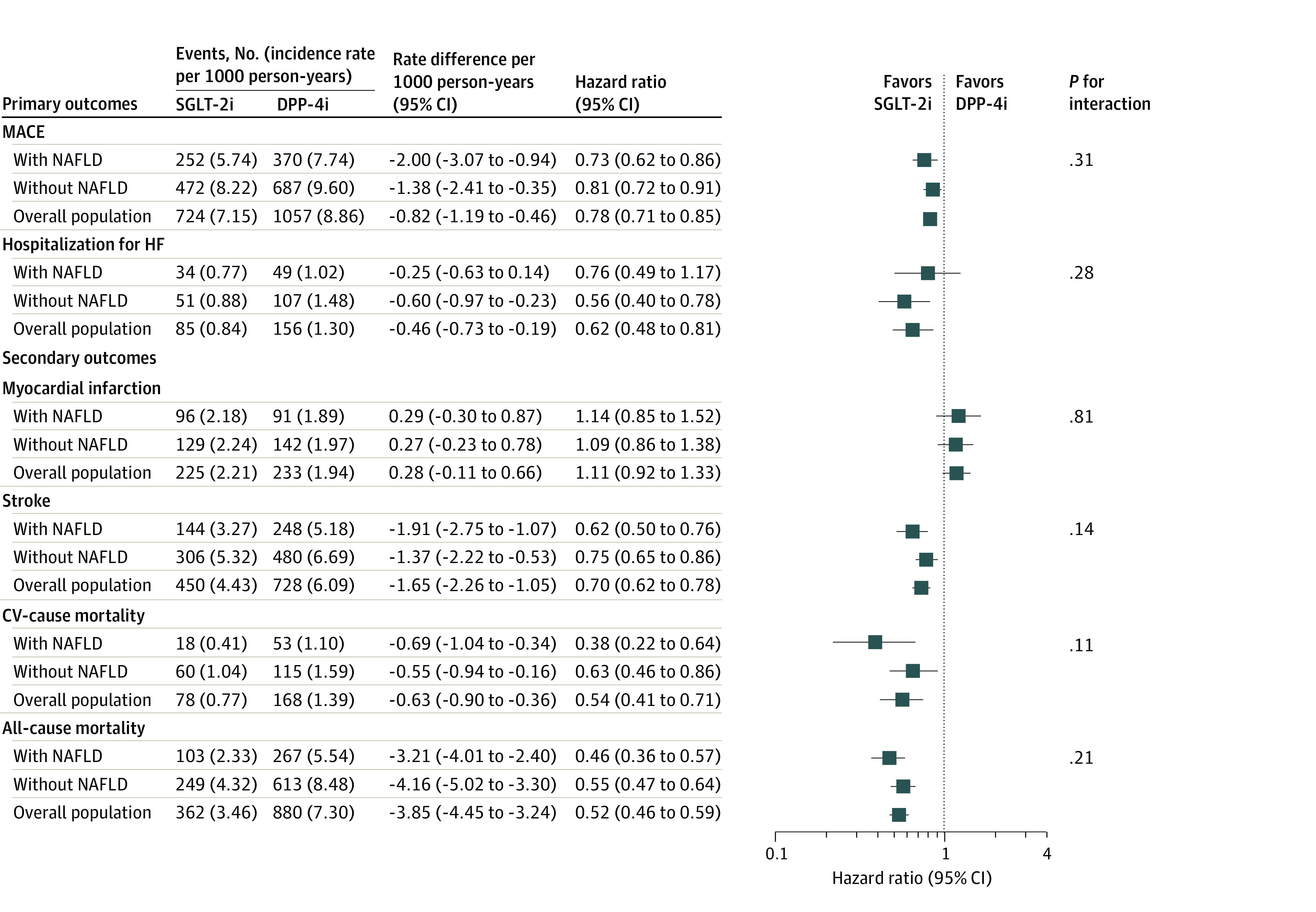
Outcomes for the 1:1 Propensity Score–Matched Cohort of New Users of Sodium Glucose Cotransporter-2 Inhibitors (SGLT-2i) and New Users of Dipeptidyl Peptidase 4 Inhibitors (DPP-4i), by Nonalcoholic Fatty Liver Disease (NAFLD) Status CV indicates cardiovascular; HF, heart failure; and MACE, major adverse cardiovascular events.

**Figure 2.**
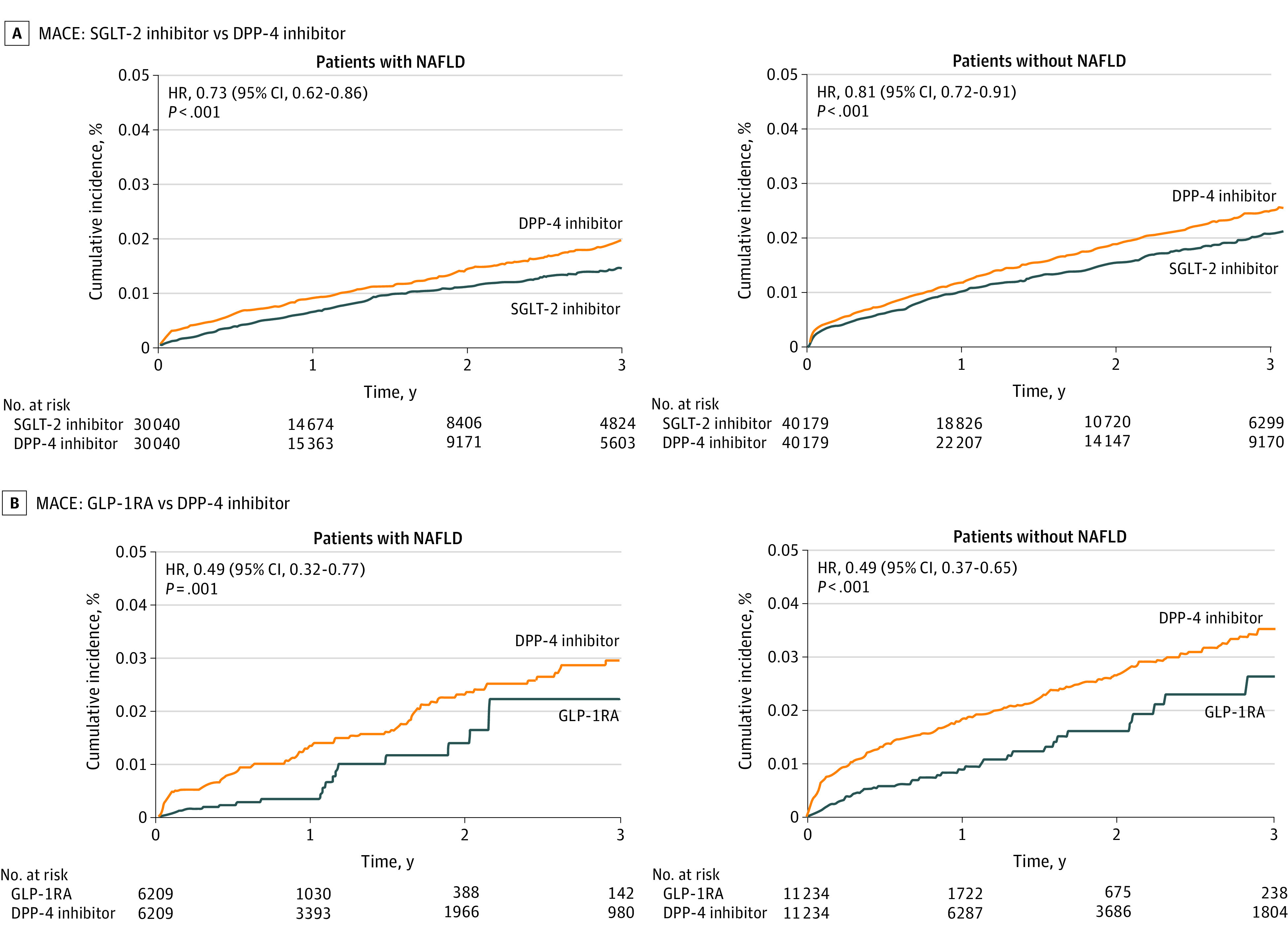
Cumulative Incidence of Major Adverse Cardiovascular Events (MACE) and Hospitalization for Heart Failure (HHF) Among Patients With Diabetes, by Nonalcoholic Fatty Liver Disease (NAFLD) Status DPP-4 indicates dipeptidyl peptidase 4; GLP-1RA, glucagon-like peptide-1 receptor agonists; HR, hazard ratio; and SGLT-2, sodium glucose cotransporter-2.

In the overall population, the risk of MACE was lower for patients using SGLT-2is compared with those using DPP-4is (HR, 0.78 [95% CI, 0.71 to 0.85]; RD, −0.82 [95% CI, −1.19 to −0.46]). The results remained consistent when stratified on NAFLD status, with no evidence of significant effect modification (with NAFLD: HR, 0.73 [95% CI, 0.62 to 0.86]; without NAFLD: HR, 0.81 [95% CI, 0.72 to 0.91]; *P *for interaction = .31). SGLT-2i showed a decreased risk of HHF in the overall population (HR, 0.62 [95% CI, 0.48 to 0.81]; RD, −0.46 [95% CI, −0.73 to −0.19]). Although not statistically significant, the point estimate in HHF was lower among patients with NAFLD group, while that of the without NAFLD group remained consistent with the overall population (with NAFLD: HR, 0.76 [95% CI, 0.49 to 1.17]; without NAFLD: HR, 0.56 [95% CI, 0.40 to 0.78]; *P *for interaction = .28). No meaningful differences were observed in the secondary effectiveness outcomes (Figure 1). There was no significant difference between the subgroups in the cumulative incidence curves of MACE; however, differences were observed in HHF (Figure 2). The findings of our sensitivity analyses were in accordance with the main findings, thus reinforcing the robustness and reliability of the results of this study (eTables 8-14 in Supplement 1). In the analysis of safety outcomes, we did not observe any safety differences between presence or absence of NAFLD (eFigure 3 in Supplement 1).

### GLP-1RA vs DPP-4i

The overall cohort of patients treated with GLP-1RAs vs DPP-4is included 234 836 patients with NAFLD and 546 568 patients without NAFLD (eFigure 2 in Supplement 1). Overall, the mean (SD) age was 61.9 (10.7) years and 56.8% of patients were male (eTable 7 in Supplement 1). After 1:1 PS matching, mean (SD) age was 59.5 (10.5) years, and 17 894 patients (51.3%) were male among the overall population (Table 2). Before matching, new users of GLP-1RA were younger, more likely to have dyslipidemia, and less likely to have received antidiabetic medications, such as metformin and sulfonylurea. After matching, an overall balance was achieved between the 2 exposure groups for all baseline covariates included in the PS estimation model (Table 2 and eTable 7 in Supplement 1). Mean (SD) follow-up durations overall were 1.06 (1.16) years for MACE (GLP-1RA, 0.6 [0.7] years; DPP-4i, 1.6 [1.3] years), and 1.07 (1.17) years for HHF (GLP-1RA, 0.6 [0.7] years; DPP-4i, 1.6 [1.3] years).

**Table 2.** Baseline Characteristics of 1:1 Propensity Score–Matched Patients Initiating GLP-1RA vs DPP-4i Overall and Across NAFLD Status

Characteristic	Patients with NAFLD			Patients without NAFLD			Overall population		
	GLP-1RA, No. (%)	DPP-4i, No. (%)	SMD	GLP-1RA, No. (%)	DPP-4i, No. (%)	SMD	GLP-1RA, No. (%)	DPP-4i, No. (%)	SMD
Patients, No.	6209	6209	NA	11 234	11 234	NA	17 443	17 443	NA
Fatty liver index, mean (SD)	79.3 (11.4)	77.8 (10.9)	0.002	30.1 (16.4)	29.5 (16.3)	0.021	47.6 (27.8)	46.7 (27.3)	0.009
Age, mean (SD), y	56.4 (10.3)	56.3 (10.4)	0.010	61.2 (10.1)	61.3 (10.4)	−0.011	59.5 (10.4)	59.5 (10.7)	−0.004
Sex									
Male	3585 (57.7)	3565 (57.4)	−0.007	5364 (47.7)	5380 (47.9)	0.003	8949 (51.3)	8945 (51.3)	0.000
Female	2624 (42.3)	2644 (42.6)		5870 (52.3)	5854 (52.1)		8494 (48.7)	8498 (48.7)	
Calendar year									
2013	66 (1.1)	59 (1.0)	0.149	36 (0.3)	48 (0.4)	0.031	102 (0.6)	107 (0.6)	0.047
2014	26 (0.4)	38 (0.6)		20 (0.2)	23 (0.2)		46 (0.3)	61 (0.3)	
2015	82 (1.3)	87 (1.4)		59 (0.5)	66 (0.6)		141 (0.8)	153 (0.9)	
2016	534 (8.6)	512 (8.2)		830 (7.4)	836 (7.4)		1364 (7.8)	1348 (7.7)	
2017	1307 (21.1)	1344 (21.6)		2425 (21.6)	2400 (21.4)		3732 (21.4)	3744 (21.5)	
2018	1422 (22.9)	1421 (22.9)		2754 (24.5)	2784 (24.8)		4176 (23.9)	4205 (24.1)	
2019	1564 (25.2)	1532 (24.7)		2843 (25.3)	2804 (25.0)		4407 (25.3)	4336 (24.9)	
2020	1208 (19.5)	1216 (19.6)		2267 (20.2)	2273 (20.2)		3475 (19.9)	3489 (20.0)	
Health care use^a^									
Inpatient hospitalizations, No.									
0	4501 (72.5)	4501 (72.5)	−0.016	7917 (70.5)	7796 (69.4)	0.023	12 418 (71.2)	12 297 (70.5)	0.023
1-2	1456 (23.4)	1479 (23.8)		2879 (25.6)	3014 (26.8)		4335 (24.9)	4493 (25.8)	
≥3	252 (4.1)	229 (3.7)		438 (3.9)	424 (3.8)		690 (4.0)	653 (3.7)	
Physician visits, No.									
0-2	53 (0.9)	66 (1.1)	0.044	55 (0.5)	66 (0.6)	0.142	108 (0.6)	132 (0.8)	0.056
3-5	163 (2.6)	161 (2.6)		171 (1.5)	198 (1.8)		334 (1.9)	359 (2.1)	
≥6	5993 (96.5)	5982 (96.3)		11 008 (98)	10 970 (97.6)		17 001 (97.5)	16 952 (97.2)	
Comorbidities^a^									
Dyslipidemia	3023 (48.7)	3039 (48.9)	−0.005	5794 (51.6)	5816 (51.8)	−0.004	8817 (50.5)	8855 (50.8)	−0.004
Hypertension	3552 (57.2)	3543 (57.1)	0.003	5090 (45.3)	5065 (45.1)	0.004	8642 (49.5)	8608 (49.3)	0.004
Atrial fibrillation	93 (1.5)	97 (1.6)	−0.005	174 (1.5)	192 (1.7)	−0.013	267 (1.5)	289 (1.7)	−0.010
Heart failure	35 (0.6)	34 (0.5)	0.002	44 (0.4)	38 (0.3)	0.009	79 (0.5)	72 (0.4)	0.006
Liver cirrhosis	209 (3.4)	208 (3.3)	0.001	348 (3.1)	350 (3.1)	−0.001	557 (3.2)	558 (3.2)	−0.000
Chronic kidney disease	34 (0.5)	36 (0.6)	−0.004	121 (1.1)	125 (1.1)	−0.003	155 (0.9)	161 (0.9)	−0.004
Dementia	349 (5.6)	359 (5.8)	−0.007	654 (5.8)	662 (5.9)	−0.003	1003 (5.8)	1021 (5.9)	−0.004
Depression	195 (3.1)	189 (3.0)	0.006	360 (3.2)	339 (3.0)	0.011	555 (3.2)	528 (3.0)	0.009
Hypothyroidism	47 (0.8)	41 (0.7)	0.012	112 (1.0)	106 (0.9)	0.005	159 (0.9)	147 (0.8)	0.007
Hyperthyroidism	136 (2.2)	150 (2.4)	−0.015	220 (2.0)	220 (2.0)	0.000	356 (2.0)	370 (2.1)	−0.006
Gallbladder disease	342 (5.5)	393 (6.3)	−0.035	667 (5.9)	657 (5.8)	0.004	1009 (5.8)	1050 (6.0)	−0.010
COPD	458 (7.4)	459 (7.4)	−0.001	1047 (9.3)	1098 (9.8)	−0.015	1505 (8.6)	1557 (8.9)	−0.011
Comedication^a^									
Acetaminophen	4116 (66.3)	4090 (65.9)	0.009	7385 (65.7)	7352 (65.4)	0.006	11 501 (65.9)	11 442 (65.6)	0.007
RAS inhibitors	4238 (68.3)	4184 (67.4)	0.019	5847 (52.0)	5809 (51.7)	0.007	10 085 (57.8)	9993 (57.3)	0.011
CCB	2881 (46.4)	2861 (46.1)	0.006	3789 (33.7)	3734 (33.2)	0.010	6670 (38.2)	6595 (37.8)	0.009
β-Blockers	1353 (21.8)	1311 (21.1)	0.016	1939 (17.3)	1989 (17.7)	−0.012	3292 (18.9)	3300 (18.9)	−0.001
Diuretics	1922 (31.0)	1935 (31.2)	−0.005	2506 (22.3)	2530 (22.5)	−0.005	4428 (25.4)	4465 (25.6)	−0.005
Systemic antibiotics	4413 (71.1)	4394 (70.8)	0.007	7952 (70.8)	7995 (71.2)	−0.008	12 365 (70.9)	12 389 (71.0)	−0.003
Oral anticoagulants	126 (2.0)	127 (2.0)	−0.001	228 (2.0)	225 (2.0)	0.002	354 (2.0)	352 (2.0)	0.001
Oral antiplatelets	2469 (39.8)	2473 (39.8)	−0.001	5021 (44.7)	5151 (45.9)	−0.023	7490 (42.9)	7624 (43.7)	−0.016
NSAIDs	4160 (67.0)	4194 (67.5)	−0.012	7580 (67.5)	7589 (67.6)	−0.002	11 740 (67.3)	11 783 (67.6)	−0.005
Opioids	816 (13.1)	804 (12.9)	0.006	1476 (13.1)	1495 (13.3)	−0.005	2292 (13.1)	2299 (13.2)	−0.001
Systemic corticosteroids	3149 (50.7)	3090 (49.8)	0.019	5934 (52.8)	5958 (53.0)	−0.004	9083 (52.1)	9048 (51.9)	0.004
Statins	4825 (77.7)	4851 (78.1)	−0.010	8869 (78.9)	8895 (79.2)	−0.006	13 694 (78.5)	13 746 (78.8)	−0.007
Other lipid-lowering agents	1958 (31.5)	1954 (31.5)	0.001	2624 (23.4)	2668 (23.7)	−0.009	4582 (26.3)	4622 (26.5)	−0.005
Vitamin E	800 (12.9)	787 (12.7)	0.006	976 (8.7)	988 (8.8)	−0.004	1776 (10.2)	1775 (10.2)	0.000
Nitrates	379 (6.1)	386 (6.2)	−0.005	803 (7.1)	831 (7.4)	−0.010	1182 (6.8)	1217 (7.0)	−0.008
Antidiabetic drugs use^a^									
Insulin	1990 (32.1)	1973 (31.8)	0.006	4667 (41.5)	4720 (42.0)	−0.010	6657 (38.2)	6693 (38.4)	−0.004
α-Glucosidase inhibitors	157 (2.5)	148 (2.4)	0.009	386 (3.4)	418 (3.7)	−0.015	543 (3.1)	566 (3.2)	−0.008
Meglitinides	53 (0.9)	58 (0.9)	−0.009	158 (1.4)	164 (1.5)	−0.004	211 (1.2)	222 (1.3)	−0.006
Metformin	5568 (89.7)	5580 (89.9)	−0.006	10 096 (89.9)	10 127 (90.1)	−0.009	15 664 (89.8)	15 707 (90.0)	−0.008
Sulfonylureas	4249 (68.4)	4177 (67.3)	0.025	7796 (69.4)	7850 (69.9)	−0.010	12 045 (69.1)	12 027 (69.0)	0.002
Thiazolidinediones	1142 (18.4)	1165 (18.8)	−0.010	2305 (20.5)	2332 (20.8)	−0.006	3447 (19.8)	3497 (20.0)	−0.007
Level of antidiabetic treatments^b^									
1	292 (4.7)	290 (4.7)	0.022	273 (2.4)	253 (2.3)	0.023	565 (3.2)	543 (3.1)	0.032
2	3927 (63.2)	3946 (63.6)		6294 (56.0)	6261 (55.7)		10 221 (58.6)	10 207 (58.5)	
3	1990 (32.1)	1973 (31.8)		4667 (41.5)	4720 (42.0)		6657 (38.2)	6693 (38.4)	
Diabetic complications^a^									
Retinopathy	596 (9.6)	611 (9.8)	−0.008	1065 (9.5)	1040 (9.3)	0.008	1661 (9.5)	1651 (9.5)	0.002
Neuropathy	1201 (19.3)	1202 (19.4)	0.000	2753 (24.5)	2835 (25.2)	−0.017	3954 (22.7)	4037 (23.1)	−0.011
Nephropathy	1605 (25.8)	1602 (25.8)	0.001	4088 (36.4)	4101 (36.5)	−0.002	5693 (32.6)	5703 (32.7)	−0.001
CCI groups^a^									
0	741 (11.9)	725 (11.7)	0.044	1176 (10.5)	1138 (10.1)	0.038	1917 (11.0)	1863 (10.7)	0.046
1-2	2547 (41.0)	2523 (40.6)		4136 (36.8)	4122 (36.7)		6683 (38.3)	6645 (38.1)	
≥3	2921 (47.0)	2961 (47.7)		5922 (52.7)	5974 (53.2)		8843 (50.7)	8935 (51.2)	
Health examination data, mean (SD)									
BMI	29.9 (4.2)	29.3 (3.7)	0.010	24.6 (2.8)	24.4 (2.7)	0.005	26.7 (4.4)	26.2 (3.9)	0.010
WC, cm	98.4 (9.2)	95.6 (8.3)	0.005	84.2 (7.5)	83.4 (7.3)	0.010	89.3 (10.6)	87.8 (9.7)	0.005
Total cholesterol, mg/dL	179.5 (48.7)	195.3 (51.8)	0.095	163.3 (41.8)	175.5 (44.7)	0.110	169.1 (45.1)	182.6 (48.3)	0.005
HDL cholesterol, mg/dL	46.8 (13.6)	48.2 (12.6)	0.010	50.3 (12.9)	52.2 (13.4)	0.005	49.0 (13.3)	50.8 (13.3)	0.010
LDL cholesterol, mg/dL	90.0 (42.4)	101.2 (50.1)	0.010	87.2 (37.1)	97.0 (41.1)	0.010	88.2 (39.0)	98.5 (44.5)	0.090
TG, mg/dL	237.1 (204.2)	258.3 (209.1)	0.005	130.1 (69.6)	132.9 (75.7)	0.010	168.2 (143.5)	177.5 (151.2)	0.085
Serum creatinine, mg/dL	0.9 (0.4)	0.9 (1.3)	0.005	0.9 (0.4)	0.9 (0.5)	0.005	0.9 (0.4)	0.9 (0.9)	0.015
AST, IU/L	37.3 (26.6)	36.5 (33.1)	0.005	26.0 (16.3)	25.9 (15.1)	0.005	30.0 (21.3)	29.7 (23.7)	0.085
ALT, IU/L	42.8 (30.6)	41.6 (32.1)	0.003	26.9 (18.6)	26.2 (17.1)	0.035	32.6 (24.8)	31.7 (24.6)	0.003
GGT, U/L	71.1 (76.2)	81.8 (94.9)	0.123	30.1 (30.7)	31.6 (32.0)	0.013	44.7 (55.3)	49.5 (66.7)	0.008
GFR, mL/min/1.73 m^2^	88.4 (32.3)	88.1 (33.8)	0.005	86.3 (26.6)	85.9 (27.3)	0.008	87.1 (28.7)	86.7 (29.8)	0.005
FBS, mg/dL	167.0 (61.9)	158.9 (33.8)	0.212	158.0 (61.4)	147.3 (56.5)	0.005	161.2 (61.7)	151.4 (57.5)	0.098

Abbreviations: ALT, serum alanine transaminase; AST, serum aspartate transaminase; BMI, body mass index (calculated as weight in kilograms divided by height in meters squared); CCB, calcium channel blocker; CCI, Charlson Comorbidity Index; COPD, chronic obstructive pulmonary disease; DPP-4i, dipeptidyl peptidase 4 inhibitor; FBG, fasting blood glucose level; GFR, glomerular filtration rate; GGT, γ-glutamyl transferase; GLP-1RA, glucagon-like peptide-1 receptor agonist; HDL, high-density lipoprotein; LDL, low-density lipoprotein; NA, not applicable; NAFLD, nonalcoholic fatty liver disease; NSAID, nonsteroidal anti-inflammatory drug; RAS, renin-angiotensin system; SMD, standardized mean difference; TG, triglyceride; WC, waist circumference.

SI conversion factors: To convert ALT, AST, and GGT to microkatals per liter, multiply by 0.0167; FBG to millimoles per liter, multiply by 0.0555; HDL, LDL, and total cholesterol to millimoles per liter, multiply by 0.0259; serum creatinine to micromoles per liter, multiply by 88.4; and TG to millimoles per liter, multiply by 0.0113.

^a^ Assessed in the years before study cohort entry.

^b^ Use of antidiabetic drugs previous 365 days before the entry date of cohort: level 1, patients who had not received any antidiabetic drugs or received only one antidiabetic; level 2, patients who had received at least 2 different classes of antidiabetic drugs without insulin; level 3, patients who had received at least 1 insulin prescription either unaccompanied or in combination with other antidiabetic drugs.

**Figure 3.**
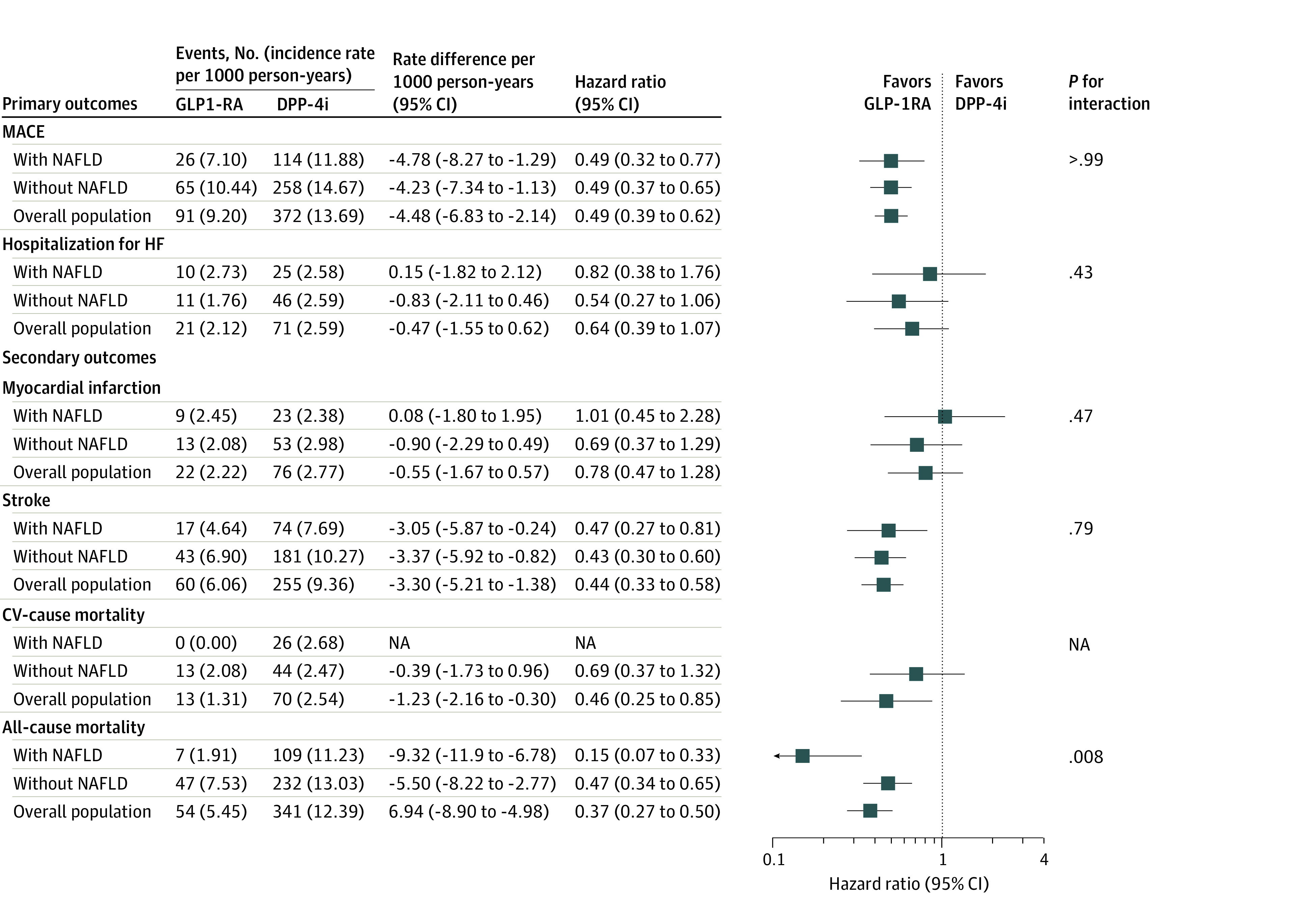
Outcomes for the 1:1 Propensity Score–Matched Cohort of New Users of Glucagon-Like Peptide-1 Receptor Agonists (GLP-1RA) vs New Users of Dipeptidyl Peptidase 4 Inhibitors (DPP-4i), by Nonalcoholic Fatty Liver Disease (NAFLD) Status CV indicates cardiovascular; HF, heart failure; MACE, major adverse cardiovascular events; and NA, not applicable.

The use of GLP-1RA was associated with a 51% decreased risk of MACE compared with DPP-4i (HR, 0.49 [95% CI, 0.32 to 0.77]; RD, −4.48 [95% CI, −6.83 to −2.14]). Consistent results were observed when stratified according to NAFLD status with no evidence of effect heterogeneity (with NAFLD: HR, 0.49 [95% CI, 0.32 to 0.77]; without NAFLD: HR, 0.49 [95% CI, 0.37 to 0.65]; *P *for interaction > .99). Meanwhile, the use of GLP-1RA was not associated with a reduced risk of HHF in the overall population (HR, 0.64 [95% CI, 0.39 to 1.07]; RD, −0.47 [95% CI, −1.55 to 0.62]; *P *for interaction = .43) and across subgroups of NAFLD status (with NAFLD: HR, 0.64 [95% CI, 0.39 to 1.07]; without NAFLD: HR, 0.54 [95% CI, 0.27 to 1.06]). The secondary effectiveness outcomes did not reveal any significant differences (Figure 3). The cumulative incidence curves of MACE and HHF were generally consistent across the subgroups (Figure 2), and the findings of the sensitivity analyses were consistent with the main findings (eTable 8-14 in Supplement 1). In safety outcomes, no differences were observed according to baseline NAFLD status (eFigure 3 in Supplement 1).

## Discussion

In this nationwide population-based cohort study, compared with the use of DPP-4i, the use of SGLT-2i was associated with a decreased risk of MACE and HHF, whereas the use of GLP-1RA was associated with a reduced risk of MACE in patients with T2D. Moreover, we observed that the effectiveness of SGLT-2i and GLP-1RA was less likely to be affected by the presence or absence of NAFLD, with no evidence of effect heterogeneity. These findings were consistent across sensitivity analyses (eAppendix 2 in Supplement 1).

### Comparison With Other Studies

To our knowledge, this is the first study to examine the comprehensive cardiovascular effectiveness of SGLT-2i in patients with NAFLD and T2D. Specifically, patients with NAFLD who received SGLT-2i exhibited a 17% reduction in MACE risk. Although there are no cardiovascular outcome trials (CVOTs) that have a focused target population of patients with NAFLD and T2D, our results for the overall population align with those of landmark CVOTs among patients with T2D, namely EMPA-REG (empagliflozin), DECLARE-TIMI 58 (dapagliflozin), and CANVAS (canagliflozin).^14,16,17^ The findings are consistent with previous observational studies comparing drugs of interest to DPP-4i. Notably, a previous study utilizing the Clinical Practice Research Datalink in the UK reported a reduced risk of MACE with SGLT-2i (HR, 0.76 [95% CI, 0.69-0.84]) and HHF (HR, 0.43 [95% CI, 0.37-0.51]).^42^ Additionally, the other study based on a US commercial health care database demonstrated a reduced risk of MACE (HR, 0.89 [95% CI, 0.68-1.17]) and HHF (HR, 0.70 [95% CI, 0.54-0.92]) among new users of canagliflozin.^43^

Several large-scale trials have shown the effects of GLP-1RA on cardiovascular and NAFLD–related outcomes. In comparison with CVOTs of GLP-1RA, such as REWIND (dulaglutide), LEADER (liraglutide), and EXSCEL (exenatide), which evaluated cardiovascular outcomes in patients with T2D, our study found a consistent reduction in MACE risk across different NAFLD statuses.^10,13,44^ In a trial of patients with NASH, semaglutide resulted in remission of NASH, demonstrating the efficacy of the potential agent in patients with NAFLD.^22^ Given that NAFLD is an independent risk factor for CVD, these findings suggest that GLP-1RAs may have enhanced cardiovascular benefits in patients with both NAFLD and T2D. However, this study did not identify effect heterogeneity according to NAFLD status, and further studies with larger populations and additional variables are warranted.

In this study, SGLT-2i, which recently expanded its indication to HF, did not differ in outcomes for HHF according to the presence or absence of NAFLD. However, the recognition of the bidirectional relationship between liver disease and HF emphasizes the critical necessity for the comprehensive management of both conditions. Supportive evidence from prior research showed that NAFLD is associated with myocardial remodeling (ie, left atrial volume index and left ventricular [LV] concentric remodeling) and functional cardiac abnormalities (ie, impaired LV systolic or diastolic function and lower e′ velocity) that have been found in many patients with HF.^45,46,47^ Recently, NAFLD has been reported to be closely associated with the development and progression of HF with preserved ejection fraction, in addition to the well-established relationship between liver disease and HF with reduced ejection fraction.^48^ Nonetheless, considering the higher mean age and greater prevalence of comorbid conditions observed among patients without NAFLD, the incidence of HHF in this group was higher than that observed in patients with NAFLD. These findings suggest that additional factors beyond the NAFLD more likely contribute to the observed variation in the association of glucose-lowering medications with the occurrence of HHF in patients with T2D. Thus, further studies with larger sample sizes and additional variables (ie, ejection fraction, lifestyle factors) are required to confirm these findings.

### Strengths and Limitations

The main strength of this study is its use of a large, nationwide data source with comprehensive health care data. Our study broadens the results of existing landmark CVOTs by reporting similar effectiveness of SGLT-2i and GLP-1RA in patients with NAFLD, a metabolically vulnerable population, compared with those without NAFLD. The results of this study also contribute to identify the safety profiles of these novel glucose-lowering medications, providing a valuable resource to assist in the development of appropriate glycemic control strategies for patients with NAFLD (eAppendix 3 in Supplement 1). Our findings also align with the current treatment guidelines that favor the use of GLP-1RA in this population and further demonstrate the effectiveness of SGLT-2i, which is known to be effective in NAFLD and liver steatosis.

The present study also has some limitations. First, as with all observational studies, we cannot rule out the possibility of residual confounding factors owing to unmeasured factors. We were unable to obtain data on important risk factors for liver disease such as alcohol use, smoking status, and dietary information.^49^ Second, although NAFLD was defined using previously validated algorithms, the results of this study may not be free from potential misclassification of NAFLD status. In particular, insulin resistance or insulin deficiency in patients with T2D can affect triglyceride levels through altered lipoprotein lipase function, which has likelihood of decrease accuracy of FLI. Nevertheless, our findings were consistent across several sensitivity analyses that used alternative definitions of NAFLD, including the hepatic steatosis index. However, future studies using abdominal ultrasonography or liver biopsy, which are considered the criterion standards for NAFLD diagnosis, may further elucidate this association in patients with NAFLD. Third, the lack of comprehensive information on NAFLD severity and baseline laboratory data such as hemoglobin A_1c_ levels or Child-Pugh scores may affect results of our findings. Also, our study may not have included metabolic changes in association with outcomes due to changes in NAFLD status. Fourth, potential bias from time lag between the health examination and cohort entry may exist. Nevertheless, our sensitivity analyses, which specifically included patients who underwent the health examination within a year before cohort entry, yielded findings consistent with the primary results. Furthermore, considering that the bias arising from the duration between health examination and cohort entry is expected to affect both groups nondifferentially. Thus, it is less likely to have influence the results of our study. Fifth, GLP-1RAs in our study were restricted to albiglutide, dulaglutide, exenatide, and lixisenatide, primarily due to reimbursement considerations in South Korea. Notably, the absence of liraglutide and semaglutide, which have growing usage owing to their established clinical effectiveness, warrants caution when interpreting the findings.

## Conclusions

This population-based cohort study found that GLP-1RA and SGLT-2i therapy were associated with reduced risk of MACE in patients with T2D and across baseline NAFLD status. Moreover, SGLT-2is were associated with reduced risk of HHF. These results support the current guidelines that recommend GLP-1RA as the first-line of therapy for patients with T2D and NAFLD. Furthermore, this study highlights the potential of SGLT-2i as a promising option for cardiovascular disease prevention regardless of NAFLD status.

## Notes

Supplement 1. **eTable 1.** Target Trial Emulation Summary **eTable 2.** Baseline Characteristics Difference Between Patients With Diabetes Who Had Health Examination Record vs Patients With Diabetes **eTable 3.** Duration Between the Latest Health Examination Date and Cohort Entry Date **eTable 4.** Definitions of Outcomes **eTable 5.** Definitions of Conditions **eTable 6.** Baseline Characteristics Before 1:1 Propensity Score Matching: Patients Initiating SGLT-2 Inhibitors vs DPP-4 Inhibitors Overall and Across NAFLD Status **eTable 7.** Baseline Characteristics Before 1:1 Propensity Score Matching: Patients Initiating GLP-1RAs vs DPP-4 Inhibitors Overall and Across NAFLD Status **eTable 8.** Sensitivity Analyses of Effectiveness Outcomes for the 1:1 Propensity Score–Matched Cohort of New Users of Sodium-Glucose Cotransporter-2 Inhibitors (SGLT-2i) or Glucagon-Like Peptide-1 Receptor Agonists (GLP-1RA) and New Users of Dipeptidyl Peptidase-4 Inhibitors (DPP-4i), by Non-Alcoholic Fatty Liver Disease (NAFLD) Status: Intention-to-Treat Analysis **eTable 9.** Sensitivity Analyses of Effectiveness Outcomes for the 1:1 Propensity Score–Matched Cohort of New Users of Sodium-Glucose Cotransporter-2 Inhibitors (SGLT-2i) or Glucagon-Like Peptide-1 Receptor Agonists (GLP-1RA) and New Users of Dipeptidyl Peptidase-4 Inhibitors (DPP-4i), by Non-Alcoholic Fatty Liver Disease (NAFLD) Status: Varying Grace Period to 90 Days **eTable 10.** Sensitivity Analyses of Effectiveness Outcomes for the 1:1 Propensity Score–Matched Cohort of New Users of Sodium-Glucose Cotransporter-2 Inhibitors (SGLT-2i) or Glucagon-Like Peptide-1 Receptor Agonists (GLP-1RA) and New Users of Dipeptidyl Peptidase-4 Inhibitors (DPP-4i), by Non-Alcoholic Fatty Liver Disease (NAFLD) Status: Varying Grace Period to 45 Days **eTable 11.** Sensitivity Analyses of Effectiveness Outcomes for the 1:1 Propensity Score–Matched Cohort of New Users of Sodium-Glucose Cotransporter-2 Inhibitors (SGLT-2i) or Glucagon-Like Peptide-1 Receptor Agonists (GLP-1RA) and New Users of Dipeptidyl Peptidase-4 Inhibitors (DPP-4i), by Non-Alcoholic Fatty Liver Disease (NAFLD) Status: Restricted Cohort Within 1 Year of Fatty Liver Index **eTable 12.** Sensitivity Analyses of Effectiveness Outcomes for the 1:1 Propensity Score–Matched Cohort of New Users of Sodium-Glucose Cotransporter-2 Inhibitors (SGLT-2i) or Glucagon-Like Peptide-1 Receptor Agonists (GLP-1RA) and New Users of Dipeptidyl Peptidase-4 Inhibitors (DPP-4i), by Non-Alcoholic Fatty Liver Disease (NAFLD) Status: Using Alternative NAFLD Definition; Hepatic Steatosis Index **eTable 13.** Sensitivity Analyses of Effectiveness Outcomes for the 1:1 Propensity Score–Matched Cohort of New Users of Sodium-Glucose Cotransporter-2 Inhibitors (SGLT-2i) or Glucagon-Like Peptide-1 Receptor Agonists (GLP-1RA) and New Users of Dipeptidyl Peptidase-4 Inhibitors (DPP-4i), by Non-Alcoholic Fatty Liver Disease (NAFLD) Status: Propensity Score Based Fine Stratification Within Average Treatment Estimate Among Whole Population **eTable 14.** Sensitivity Analyses of Effectiveness Outcomes for the 1:1 Propensity Score–Matched Cohort of New Users of Sodium-Glucose Cotransporter-2 Inhibitors (SGLT-2i) or Glucagon-Like Peptide-1 Receptor Agonists (GLP-1RA) and New Users of Dipeptidyl Peptidase-4 Inhibitors (DPP-4i), by Non-Alcoholic Fatty Liver Disease (NAFLD) Status: Treating All-Cause Death as Competing Event **eAppendix 1.** Definitions of Proxy Indicator for NAFLD Definition **eAppendix 2.** Description of Sensitivity Analyses **eAppendix 3.** Description of Exploratory Analyses **eFigure 1.** Flowchart of Study Population Selection Among New Users of SGLT-2 Inhibitors vs DPP-4 Inhibitors With Varying NAFLD Status **eFigure 2.** Flowchart of Study Population Selection Among New Users of GLP-1RAs vs DPP-4 Inhibitors With Varying NAFLD Status **eFigure 3.** Results of Exploratory Analyses

Supplement 2.
Data Sharing Statement
