# Supplementary material for: Outcomes of SGLT-2i and GLP-1RA Therapy Among Patients With Type 2 Diabetes and Varying NAFLD Status
Source: JAMA Netw Open. 2023 Dec 28;6(12):e2349856. doi: 10.1001/jamanetworkopen.2023.49856 (PMC10755620; doi:10.1001/jamanetworkopen.2023.49856)
Supplement: Supplement 1. — eTable 1. Target Trial Emulation Summary eTable 2. Baseline Characteristics Difference Between Patients With Diabetes Who Had Health Examination Record vs Patients With Diabetes eTable 3. Duration Between the Latest Health Examination Date and Cohort Entry Date eTable 4. Definitions of Outcomes eTable 5. Definitions of Conditions eTable 6. Baseline Characteristics Before 1:1 Propensity Score Matching: Patients Initiating SGLT-2 Inhibitors vs DPP-4 Inhibitors Overall and Across NAFLD Status eTable 7. Baseline Characteristics Before 1:1 Propensity Score Matching: Patients Initiating GLP-1RAs vs DPP-4 Inhibitors Overall and Across NAFLD Status eTable 8. Sensitivity Analyses of Effectiveness Outcomes for the 1:1 Propensity Score–Matched Cohort of New Users of Sodium-Glucose Cotransporter-2 Inhibitors (SGLT-2i) or Glucagon-Like Peptide-1 Receptor Agonists (GLP-1RA) and New Users of Dipeptidyl Peptidase-4 Inhibitors (DPP-4i), by Non-Alcoholic Fatty Liver Disease (NAFLD) Status: Intention-to-Treat Analysis eTable 9. Sensitivity Analyses of Effectiveness Outcomes for the 1:1 Propensity Score–Matched Cohort of New Users of Sodium-Glucose Cotransporter-2 Inhibitors (SGLT-2i) or Glucagon-Like Peptide-1 Receptor Agonists (GLP-1RA) and New Users of Dipeptidyl Peptidase-4 Inhibitors (DPP-4i), by Non-Alcoholic Fatty Liver Disease (NAFLD) Status: Varying Grace Period to 90 Days eTable 10. Sensitivity Analyses of Effectiveness Outcomes for the 1:1 Propensity Score–Matched Cohort of New Users of Sodium-Glucose Cotransporter-2 Inhibitors (SGLT-2i) or Glucagon-Like Peptide-1 Receptor Agonists (GLP-1RA) and New Users of Dipeptidyl Peptidase-4 Inhibitors (DPP-4i), by Non-Alcoholic Fatty Liver Disease (NAFLD) Status: Varying Grace Period to 45 Days eTable 11. Sensitivity Analyses of Effectiveness Outcomes for the 1:1 Propensity Score–Matched Cohort of New Users of Sodium-Glucose Cotransporter-2 Inhibitors (SGLT-2i) or Glucagon-Like Peptide-1 Receptor Agonists (GLP-1RA) and New Users of Di [file jamanetwopen-e2349856-s001.pdf]

Supplemental Online Content

Bea S, Jeong HE, Filion KB, et al. Outcomes of SGLT-2i and GLP-1RA therapy among patients with type 2 diabetes and varying NAFLD status. *JAMA Netw Open.* 2024;7(1):e2349856. doi:10.1001/jamanetworkopen.2023.49856

eTable 1. Target Trial Emulation Summary

eTable 2. Baseline Characteristics Difference Between Patients With Diabetes Who Had Health Examination Record vs Patients With Diabetes

eTable 3. Duration Between the Latest Health Examination Date and Cohort Entry Date

eTable 4. Definitions of Outcomes

eTable 5. Definitions of Conditions

eTable 6. Baseline Characteristics Before 1:1 Propensity Score Matching: Patients Initiating SGLT-2 Inhibitors vs DPP-4 inhibitors Overall and Across NAFLD Status

eTable 7. Baseline Characteristics Before 1:1 Propensity Score Matching: Patients Initiating GLP-1RAs vs DPP-4 Inhibitors Overall and Across NAFLD Status

eTable 8. Sensitivity Analyses of Effectiveness Outcomes for the 1:1 Propensity Score–Matched Cohort of New Users of Sodium-Glucose Cotransporter-2 Inhibitors (SGLT-2i) or Glucagon-Like Peptide-1 Receptor Agonists (GLP-1RA) and New Users of Dipeptidyl Peptidase-4 Inhibitors (DPP-4i), by Non-Alcoholic Fatty Liver Disease (NAFLD) Status: Intention-to-Treat Analysis

eTable 9. Sensitivity Analyses of Effectiveness Outcomes for the 1:1 Propensity Score–Matched Cohort of New Users of Sodium-Glucose Cotransporter-2 Inhibitors (SGLT-2i) or Glucagon-Like Peptide-1 Receptor Agonists (GLP-1RA) and New Users of Dipeptidyl Peptidase-4 Inhibitors (DPP-4i), by Non-Alcoholic Fatty Liver Disease (NAFLD) Status: Varying Grace Period to 90 Days

eTable 10. Sensitivity Analyses of Effectiveness Outcomes for the 1:1 Propensity Score–Matched Cohort of New Users of Sodium-Glucose Cotransporter-2 Inhibitors (SGLT-2i) or Glucagon-Like Peptide-1 Receptor Agonists (GLP-1RA) and New Users of Dipeptidyl Peptidase-4 Inhibitors (DPP-4i), by Non-Alcoholic Fatty Liver Disease (NAFLD) Status: Varying Grace Period to 45 Days

eTable 11. Sensitivity Analyses of Effectiveness Outcomes for the 1:1 Propensity Score–Matched Cohort of New Users of Sodium-Glucose Cotransporter-2 Inhibitors (SGLT-2i) or Glucagon-Like Peptide-1 Receptor Agonists (GLP-1RA) and New Users of Dipeptidyl Peptidase-4 Inhibitors (DPP-4i), by Non-Alcoholic Fatty Liver Disease (NAFLD) Status: Restricted Cohort Within 1 Year of Fatty Liver Index

eTable 12. Sensitivity Analyses of Effectiveness Outcomes for the 1:1 Propensity Score–Matched Cohort of New Users of Sodium-Glucose Cotransporter-2 Inhibitors (SGLT-2i) or Glucagon-Like Peptide-1 Receptor Agonists (GLP-1RA) and New Users of Dipeptidyl Peptidase-4 Inhibitors (DPP-4i), by Non-Alcoholic Fatty Liver Disease (NAFLD) Status: Using Alternative NAFLD Definition; Hepatic Steatosis Index

eTable 13. Sensitivity Analyses of Effectiveness Outcomes for the 1:1 Propensity Score–Matched Cohort of New Users of Sodium-Glucose Cotransporter-2 Inhibitors (SGLT-2i) or Glucagon-Like Peptide-1 Receptor Agonists (GLP-1RA) and New Users of Dipeptidyl Peptidase-4 Inhibitors (DPP-4i), by Non-Alcoholic Fatty Liver Disease (NAFLD) Status: Propensity Score Based Fine Stratification Within Average Treatment Estimate Among Whole Population

eTable 14. Sensitivity Analyses of Effectiveness Outcomes for the 1:1 Propensity Score–Matched Cohort of New Users of Sodium-Glucose Cotransporter-2 Inhibitors (SGLT-2i) or Glucagon-Like Peptide-1 Receptor Agonists (GLP-1RA) and New Users of Dipeptidyl Peptidase-4 Inhibitors (DPP-

© 2023 Bea S et al. *JAMA Network Open.*

4i), by Non-Alcoholic Fatty Liver Disease (NAFLD) Status: Treating All-Cause Death as Competing Event

eAppendix 1. Definitions of Proxy Indicator for NAFLD Definition

eAppendix 2. Description of Sensitivity Analyses

eAppendix 3. Description of Exploratory Analyses

eFigure 1. Flowchart of Study Population Selection Among New Users of SGLT-2 Inhibitors vs DPP-4 Inhibitors With Varying NAFLD Status

eFigure 2. Flowchart of Study Population Selection Among New Users of GLP-1RAs vs DPP-4 Inhibitors With Varying NAFLD Status

eFigure 3. Results of Exploratory Analyses

This supplemental material has been provided by the authors to give readers additional information about their work.

© 2023 Bea S et al. *JAMA Network Open.*

eTable 1. Target Trial Emulation Summary

Component	Hypothetical Target Trial	Emulated trial using real-world database
**Aim**	To investigate the effectiveness of these novel antidiabetics among patients with T2D stratified by NAFLD status.	Same
**Setting**	Korean national health insurance service (NHIS) beneficiaries. Pragmatic large simplified trial in the context of real-world routine clinical practice.	Same
**Eligibility**	1. Study antidiabetic drug (SGLT-2i vs DPP-4i or SGLT-2i vs GLP-1RA) prescription fill between 1/1/2013-12/31/2020 for NHIS beneficiaries ≥40 years of age<br>2. New-users of study antidiabetic drug<br>3. Type 2 diabetes<br>4. With or without NAFLD<br>5. History of health examination<br>6. No history of end-stage renal disease or dialysis (exclusion criteria)<br>7. No history of liver-related diseases (exclusion criteria)	Same (FLI as a surrogate indicator of NAFLD, which is extensively validated as an alternative metric to liver image screening in large population-based epidemiologic studies. Moreover, the FLI has been previously validated in the Korean population, with a PPV of 89%.)
**Treatment strategies**	1. Receiving SGLT-2i treatment<br>Versus Receiving DPP-4i treatment<br>2. Receiving SGLT-2i treatment<br>Versus Receiving GLP-1RA treatment	Same
**Treatment assignment**	Patients are randomly assigned to either treatment strategy	Propensity score methods were applied to generate similar probability of receiving treatment strategy
**Follow-up initiation**	Date of randomization	Date of first prescription of study antidiabetic drugs
**Follow-up termination**	Follow-up ends on the first of:<br>1. Occurrence of a study outcome<br>2. Censoring due to discontinuation (or switching) of the cohort entry-defining drug<br>3. Death<br>4. End of the study period	Same
**Primary outcomes**	1. Major adverse cardiovascular events<br>2. Hospitalization for heart failure	Same
**Secondary outcomes**	1. Hospitalization for myocardial infarction,<br>2. Hospitalization for stroke<br>3. Cardiovascular death<br>4. All-cause death	Same

© 2023 Bea S et al. *JAMA Network Open.*

**Causal contrasts**	Primary analysis: per-protocol effect<br>Sensitivity analysis: intention-to-treat effect	Same
**Statistical analysis**	All outcome measures in this study were derived from the time-to-event analysis. Commonly utilized metrics, such as ratio and difference measures, were employed to quantify the treatment effects.<br>1. Cox proportional hazard regression: Hazard ratio<br>2. Poisson regression: Rate difference	Same

Abbreviations: DPP-4i, dipeptidyl peptidase 4 inhibitors; FLI, fatty liver index; GLP-1RA, glucagon-like peptide-1 receptor agonists; NAFLD, non-alcoholic fatty liver disease; SGLT-2i, sodium glucose cotransporter 2 inhibitors; T2D, type 2 diabetes

eTable 2. Baseline Characteristics Difference Between Patients With Diabetes Who Had Health Examination Record vs Patients With Diabetes

	Cohort 1 (SGLT-2i vs DPP-4i)	Cohort 2 (GLP-1RA vs DPP-4i)
	**Standardized mean difference**	**Standardized mean difference**
**Age, years; mean (SD)**	0.012	0.005
**Male, No. (%)**	-0.014	-0.016
**Calendar year**	0.082	0.072
Inpatient hospitalizations	0.055	0.062
Number of physician visits	0.095	0.065
**Comorbidities**‡		
Dyslipidemia	0.056	0.048
Hypertension	0.024	0.013
Atrial fibrillation	-0.008	-0.009
Heart failure	-0.008	-0.010
Liver cirrhosis	-0.035	-0.036
Chronic kidney disease	-0.032	-0.031
Dementia	-0.004	-0.002
Depression	0.006	0.006
Hypothyroidism	0.002	0.001
Hyperthyrodism	0.005	0.003
Gallbladder disease	0.009	0.006
COPD	0.015	0.012
**Comedication**‡		
Acetaminophen	0.054	0.303
RAS inhibitors	0.011	0.247
CCB	-0.006	0.215
β-blockers	-0.021	0.128
Diuretics	-0.011	0.167
Systemic antibiotics	0.049	0.321
Oral anticoagulants	-0.014	0.039
Oral antiplatelets	0.004	0.193
NSAIDs	0.061	0.308
Opioids	0.012	0.102
Systemic corticosteroids	0.062	0.262
Statins	0.059	0.258
Other lipid-lowering agents	0.025	0.111
Vitamin E	-0.003	0.074
Nitrates	-0.006	0.068
**Antidiabetic drugs use**‡		
Insulin	-0.047	0.099
α-glucosidase inhibitors	-0.003	0.073
Meglitinides	-0.011	0.027
Metformin	0.051	0.313
Sulfonylureas	0.002	0.212
Thiazolidinediones	0.006	0.075
Level of antidiabetic treatments§	0.028	0. 293
**Diabetic complications**‡		
Retinopathy	-0.005	-0.008
Neuropathy	0.011	0.005
Nephropathy	0.016	0.012
**CCI groups**‡	0.051	0.050

Abbreviation: CCB, calcium channel blocker; CCI, Charlson's comorbidity index; COPD, chronic obstructive pulmonary disease; NAFLD, non-alcoholic fatty liver disease; NSAID, nonsteroidal anti-inflammatory drugs

§Use of antidiabetic drugs previous 365 days before the entry date of cohort: level 1, Patients not received any of antidiabetics or

© 2023 Bea S et al. *JAMA Network Open.*

received only one antidiabetics; level 2, Patients received ≥2 different classes of antidiabetics without insulin; level 3, Patients received ≥1 insulin either unaccompanied or in combination with other antidiabetics.
‡Assessed in the years before study cohort entry.

© 2023 Bea S et al. *JAMA Network Open*.

eTable 3. Duration Between the Latest Health Examination Date and Cohort Entry Date

**Cohort 1 (SGLT-2i vs DPP-4i)**	**Days, median (interquartile range)**
Main analysis (within 3 year prior to cohort entry)	547 (193-889)
Sensitivity analysis (within 1 year prior to cohort entry)	105 (25-232)
**Cohort 2 (GLP-1RA vs DPP-4i)**	**Days**
Main analysis (within 3 year prior to cohort entry)	490 (189-834)
Sensitivity analysis (within 1 year prior to cohort entry)	129 (35-247)

© 2023 Bea S et al. *JAMA Network Open.*

eTable 4. Definitions of Outcomes[*]

Outcome				Definitions	PPV (%)
Effectiveness	MACE	Myocardial infarction		Defined as hospitalized primary diagnosis.<br>ICD-10 codes: I21, I22.	92.0[*] (184/200)
		Stroke	Hemorrhagic stroke	Defined as hospitalized primary diagnosis.<br>ICD-10 codes: I63, I64.	90.5[*] (181/200)
			Ischemic stroke	Defined as hospitalized primary diagnosis with concomitant imaging studies of the brain or related death.<br>ICD-10 codes: I60, I61, I62.	87.5[†] (286/327)
		Cardiovascular death		Death records with cause of death ICD-10: "I"	N/A
	Hospitalization for heart failure			Defined from primary or first secondary admission diagnoses of heart failure<br>ICD-10 codes: I110, I971, I50	82.1%[†] (110/134)
Safety	Non-vertebral fracture			Defined as hospitalized the primary (e.g., most accountable) and secondary position.<br>Other outcomes were defined as ≥1 diagnosis from an inpatient setting or ≥2 independent diagnoses within 180 days from an outpatient setting; the first date was used as the event date for the latter definition.<br>ICD-10 codes: S92, T12, S82, S72, S62, S52, T10, S42, S12, T02	
	Acute kidney injury			Defined as hospitalized the primary (e.g., most accountable) and secondary position.<br>Other outcomes were defined as ≥1 diagnosis from an inpatient setting or ≥2 independent diagnoses within 180 days from an outpatient setting; the first date was used as the event date for the latter definition.<br>ICD-10 codes: N17	
	Genital infection			Defined as hospitalized the primary (e.g., most accountable) and secondary position.<br>Other outcomes were defined as ≥1 diagnosis from an inpatient setting or ≥2 independent diagnoses within 180 days from an outpatient setting; the first date was used as the event date for the latter definition.<br>ICD-10 codes: N76, N47, B373, N771, B374, N481, K404	
	Lower limb amputation			Defined as hospitalized the primary (e.g., most accountable) and secondary position.<br>Other outcomes were defined as ≥1 diagnosis from an inpatient setting or ≥2 independent diagnoses within 180 days from an outpatient setting; the first date was used as the event date for the	

© 2023 Bea S et al. *JAMA Network Open.*

		latter definition.<br>Procedure code (domestic): N0571, N0572, N0573, N0574, N0575	
	Diabetic ketoacidosis	Defined as hospitalized the primary (e.g., most accountable) and secondary position. Required records of in-hospital admission or emergency room visits on the date of outcome occurrence<br>ICD-10 codes: E111, E131, E141	
	Hypoglycemia	Defined as hospitalized the primary (e.g., most accountable) and secondary position. Required records of in-hospital admission or emergency room visits on the date of outcome occurrence<br>ICD-10 codes: E160, E161, E162, E1163, E1363, E1463	
	Pancreatitis	Defined as hospitalized the primary (e.g., most accountable) and secondary position.<br>Other outcomes were defined as ≥1 diagnosis from an inpatient setting or ≥2 independent diagnoses within 180 days from an outpatient setting; the first date was used as the event date for the latter definition.<br>ICD-10 codes: K85, K86	
	Gastrointestinal disorders	Defined as hospitalized the primary (e.g., most accountable) and secondary position.<br>Other outcomes were defined as ≥1 diagnosis from an inpatient setting or ≥2 independent diagnoses within 180 days from an outpatient setting; the first date was used as the event date for the latter definition.<br>ICD-10 codes: K90, K91, K92, K93	

N/A=not applicable.

* Validated in the study by Park, J. et al. (Park J, Kwon S, Choi E-K et al. Validation of diagnostic codes of major clinical outcomes in a National Health Insurance database. International Journal of Arrhythmia 2019;20:5.

† Validated in the study by Kim, D. et al. (Kim D, Yang P-S, You SC et al. Treatment timing and the effects of rhythm control strategy in patients with atrial fibrillation: nationwide cohort study. BMJ (Clinical research ed) 2021;373:n991.)

© 2023 Bea S et al. *JAMA Network Open.*

eTable 5. Definitions of Conditions [*]

Conditions	Comment (if applicable)[*]
**Exclusion Criteria**	
**Other liver disease**	Autoimmune liver disease, ICD-10: K743, K754, K830<br>Biliary cirrhosis, ICD-10: K744, K745<br>Budd-Chiari syndrome, ICD-10: I820, K765<br>Chronic hepatitis unspecified, ICD-10: K732, K739<br>Disorders of plasma-protein metabolism, ICD-10: E880<br>Hemochromatosis, ICD-10: E831<br>Wilson's disease, ICD-10: E830<br>Secondary/unspecified biliary cirrhosis, K744, K745
**Viral hepatitis**	Acute hepatitis B, ICD-10: B16<br>Other acute viral hepatitis, ICD-10: B17<br>Chronic viral hepatitis, ICD-10: B18<br>Unspecified viral hepatitis, ICD-10: B19
**Alcohol-related liver disease**	Alcoholic liver disease, ICD-10: K70
**End-stage renal disease**	Chronic kidney disease (stage 5), ICD-10: N185<br>Care involving dialysis, ICD-10: Z49<br>Dependence on renal dialysis, ICD-10: Z992<br>Domestic procedure code: O7020, O7061, O7062
**Covariates**	
**Demographic**	
Age	Defined at cohort entry date
Sex	Male or Female
**Comorbidities**	**ICD-10 codes**
Atrial fibrillation	I48.x Atrial fibrillation and flutter
Chronic kidney disease	N18.x Chronic kidney disease<br>N19.x Unspecified kidney failure
Chronic obstructive pulmonary disease	J42.x Unspecified chronic bronchitis<br>J43.x Emphysema<br>J44.x Other chronic obstructive pulmonary disease
Depression	F32 Depressive episode<br>F33 Recurrent depressive disorder<br>F341 Persistent mood disorders
Dementia	F01.x Vascular dementia<br>F02.x Dementia in other diseases classified elsewhere<br>F03.x Unspecified dementia<br>G30.x Alzheimer's disease<br>G31.0 Frontotemporal dementia<br>G31.82 Leigh's disease
Dyslipidemia	E78.x Disorders of lipoprotein metabolism and other lipidemia
Gallbladder disease	K563 Gallstone ileus<br>K80.x Cholelithiasis<br>K81.x Cholecystitis<br>K82.x Other diseases of gallbladder<br>K83.x Other diseases of biliary tract<br>K85.1 Biliary acute pancreatitis<br>K87.x Disorders of gallbladder, biliary tract and pancreas in diseases classified elsewhere
Heart failure	I50.x Heart failure<br>I971 Other functional disturbances following cardiac surgery (Heart failure)
Hypertension	I10.x Essential (primary) hypertension<br>I11.x Hypertensive heart disease<br>I12.x Hypertensive chronic kidney disease<br>I13.x Hypertensive heart and chronic kidney disease<br>I15.x Secondary hypertension

© 2023 Bea S et al. *JAMA Network Open.*

Hyperthyroidism	E05.x Hyperthyroidism
Hypothyroidism	E03.x Other hypothyroidism
Liver cirrhosis	K702 Alcoholic fibrosis and sclerosis of liver<br>K703 Alcoholic cirrhosis of liver<br>K704 Alcoholic hepatic failure<br>K74.x Fibrosis and cirrhosis of liver
**Comedication use**	**ATC codes**
Acetaminophen	N02BE01, N02BE05, N02BE51, N02BE71, N02AJ13
α-glucosidase inhibitors	A10BF
β-blockers	C07
Calcium channel blockers	C08, C07FB, C09BB, C09DB
Diuretics	C03A, C03DA, C07B, C07D, C03C, C03E, C03X, C07C, C08G
DPP-4i	A10BH
GLP-1RA	A10BJ
Insulin	A10A
Meglitinides	A10BX02, A10BX03, A10BX08
Metformin	A10BA02
Nitrates	C01DA
Nonsteroidal anti-inflammatory drugs	M01A
Opioids	N02A
Oral anticoagulants	B01AA (excl. B01AA03), B01AB, B01AD, B10AE, B10AF, B01AX
Oral antiplatelets	B01AF
Other lipid-lowering agents	C10AB, C10AC, C10AX
Renin angiotensin inhibitors	C09A, C09B, C09C, C09D
SGLT-2i	A10BK
Systemic corticosteroids	A07EA
Systemic antibiotics	J01
Sulfonylureas	A10BB
Statins	C10AA
Thiazolidinediones	A10BG
Vitamin E	N02A
No. of different classes of non-antidiabetic medications	Measured by drug class; assessed in the year prior to and including study cohort entry (categorized as 0-1, 2-5, or ≥6)
**Healthcare use**	
Number of inpatient hospitalizations	In a year prior to and including cohort entry (categorized as 0, 1-2, or ≥3)
Number of physician visits	Included in- and outpatient visits in a year prior to cohort entry (categorized as 0-2, 3-5, or ≥6)

Abbreviations: ATC, Anatomical Therapeutic Chemical classification code; DPP-4i, dipeptidyl peptidase 4 inhibitors; GLP-1RA, glucagon-like peptide-1 receptor agonists; ICD-10, International Classification of Diseases, 10th revision; SGLT-2i, sodium glucose cotransporter 2 inhibitors

*Unless otherwise specified, comorbidities ascertained from hospitalization or physician claims data in the three years prior to study cohort entry. Comedications and healthcare use assessed in the year prior to study cohort entry. Comorbidities were measured using ICD-10 codes, and procedures were defined using domestic codes.

© 2023 Bea S et al. *JAMA Network Open.*

eTable 6. Baseline Characteristics Before 1:1 Propensity Score Matching: Patients Initiating SGLT-2 Inhibitors vs DPP-4 inhibitors Overall and Across NAFLD Status

	With NAFLD			Without NAFLD			Overall Population		
	**SGLT-2i**	**DPP-4i**	**SMD**	**SGLT-2i**	**DPP-4i**	**SMD**	**SGLT-2i**	**DPP-4i**	**SMD**
**Number of patients**	30060	184393		40181	426493		70241	610886	
**Fatty liver index, mean (SD)**	79.3 (11.1)	77.1 (10.7)		33.4 (15.8)	30.2 (16.1)		53.1 (26.7)	44.4 (26.0)	
**Age, years; mean (SD)**	54.7 (9.6)	58.3 (10.8)	-.355	59.8 (10.1)	63.8 (10.8)	-.390	57.6 (10.2)	62.1 (11.1)	-.430
**Male, No. (%)**	20100 (66.9)	130313 (70.7)	0.082	19594 (48.8)	219842 (51.5)	0.056	39694 (56.5)	350155 (57.3)	0.016
**Calendar year**			0.454			0.431			0.445
2014	732 (2.4)	10470 (5.7)		1323 (3.3)	26203 (6.1)		2055 (2.9)	36673 (6)	
2015	2645 (8.8)	32812 (17.8)		4060 (10.1)	85615 (20.1)		6705 (9.5)	118427 (19.4)	
2016	3817 (12.7)	33870 (18.4)		5618 (14)	82141 (19.3)		9435 (13.4)	116011 (19)	
2017	5087 (16.9)	31038 (16.8)		7135 (17.8)	70961 (16.6)		12222 (17.4)	101999 (16.7)	
2018	5020 (16.7)	28181 (15.3)		6292 (15.7)	60445 (14.2)		11312 (16.1)	88626 (14.5)	
2019	6373 (21.2)	25076 (13.6)		8098 (20.2)	53465 (12.5)		14471 (20.6)	78541 (12.9)	
2020	6386 (21.2)	22946 (12.4)		7655 (19.1)	47663 (11.2)		14041 (20)	70609 (11.6)	
**Healthcare use‡**									
Inpatient hospitalizations			0.088			0.071			0.085
0	24505 (81.5)	146837 (79.6)		31829 (79.2)	326572 (76.6)		56334 (80.2)	473409 (77.5)	
1-2	5055 (16.8)	33236 (18)		7522 (18.7)	87236 (20.5)		12577 (17.9)	120472 (19.7)	
≥3	500 (1.7)	4320 (2.3)		830 (2.1)	12685 (3)		1330 (1.9)	17005 (2.8)	
Number of physician visits			0.036			0.044			0.079
0-2	1659 (5.5)	10847 (5.9)		1571 (3.9)	16991 (4)		3230 (4.6)	27838 (4.6)	
3-5	2749 (9.1)	14881 (8.1)		2568 (6.4)	23297 (5.5)		5317 (7.6)	38178 (6.2)	
≥6	25652 (85.3)	158665 (86)		36042 (89.7)	386205 (90.6)		61694 (87.8)	544870 (89.2)	
**Comorbidities‡**									
Dyslipidemia	12875 (42.8)	69687 (37.8)	0.103	17168 (42.7)	162200 (38)	0.096	30043 (42.8)	231887 (38)	0.098
Hypertension	16372 (54.5)	103098 (55.9)	-.029	18738 (46.6)	209958 (49.2)	-.052	35110 (50)	313056 (51.2)	-.025
Atrial fibrillation	405 (1.3)	2523 (1.4)	-.002	710 (1.8)	6648 (1.6)	0.016	1115 (1.6)	9171 (1.5)	0.007
Heart failure	69 (0.2)	743 (0.4)	-.031	69 (0.2)	1246 (0.3)	-.025	138 (0.2)	1989 (0.3)	-.025
Liver cirrhosis	160 (0.5)	2378 (1.3)	-.080	280 (0.7)	6323 (1.5)	-.076	440 (0.6)	8701 (1.4)	-.079
Chronic kidney disease	75 (0.2)	1255 (0.7)	-.063	254 (0.6)	5530 (1.3)	-.068	329 (0.5)	6785 (1.1)	-.073
Dementia	1189 (4)	7591 (4.1)	-.008	1805 (4.5)	21761 (5.1)	-.029	2994 (4.3)	29352 (4.8)	-.026
Depression	703 (2.3)	3284 (1.8)	0.039	1225 (3)	10924 (2.6)	0.029	1928 (2.7)	14208 (2.3)	0.027
Hypothyroidism	208 (0.7)	1036 (0.6)	0.016	442 (1.1)	4182 (1)	0.012	650 (0.9)	5218 (0.9)	0.008
Hyperthyrodism	564 (1.9)	3100 (1.7)	0.015	715 (1.8)	7218 (1.7)	0.007	1279 (1.8)	10318 (1.7)	0.010
Gallbladder disease	1341 (4.5)	10045 (5.4)	-.045	2241 (5.6)	28617 (6.7)	-.047	3582 (5.1)	38662 (6.3)	-.053
COPD	1677 (5.6)	11086 (6)	-.019	2802 (7)	32178 (7.5)	-.022	4479 (6.4)	43264 (7.1)	-.028
**Comedication‡**									
Acetaminophen	17490 (58.2)	110256 (59.8)	-.033	24838 (61.8)	271329 (63.6)	-.037	42328 (60.3)	381585 (62.5)	-.045
RAS inhibitors	16257 (54.1)	95476 (51.8)	0.046	18080 (45)	184048 (43.2)	0.037	34337 (48.9)	279524 (45.8)	0.063
CCB	12711 (42.3)	78647 (42.7)	-.007	13809 (34.4)	152365 (35.7)	-.028	26520 (37.8)	231012 (37.8)	-.001
β-blockers	5312 (17.7)	31042 (16.8)	0.022	6655 (16.6)	63514 (14.9)	0.046	11967 (17)	94556 (15.5)	0.042
Diuretics	7495 (24.9)	50176 (27.2)	-.052	8540 (21.3)	99353 (23.3)	-.049	16035 (22.8)	149529 (24.5)	-.039
Systemic antibiotics	19583 (65.1)	119966 (65.1)	0.002	26932 (67)	288201 (67.6)	-.012	46515 (66.2)	408167 (66.8)	-.013
Oral anticoagulants	415 (1.4)	2666 (1.4)	-.006	778 (1.9)	7530 (1.8)	0.013	1193 (1.7)	10196 (1.7)	0.002
Oral antiplatelets	7366 (24.5)	50266 (27.3)	-.063	12235 (30.4)	137726 (32.3)	-.040	19601 (27.9)	187992 (30.8)	-.063
NSAIDs	18236 (60.7)	112892 (61.2)	-.011	25688 (63.9)	277385 (65)	-.023	43924 (62.5)	390277 (63.9)	-.028
Opioids	2800 (9.3)	18735 (10.2)	-.028	4049 (10.1)	47981 (11.3)	-.038	6849 (9.8)	66716 (10.9)	-.038
Systemic corticosteroids	14804 (49.2)	91415 (49.6)	-.006	21270 (52.9)	227077 (53.2)	-.006	36074 (51.4)	318492 (52.1)	-.016
Statins	15563 (51.8)	86153 (46.7)	0.101	22567 (56.2)	216559 (50.8)	0.108	38130 (54.3)	302712 (49.6)	0.095
Other lipid-lowering agents	5913 (19.7)	28917 (15.7)	0.105	6300 (15.7)	46536 (10.9)	0.141	12213 (17.4)	75453 (12.4)	0.142
Vitamin E	2282 (7.6)	13251 (7.2)	0.015	2201 (5.5)	22403 (5.3)	0.010	4483 (6.4)	35654 (5.8)	0.023
Nitrates	1495 (5)	7657 (4.2)	0.039	2739 (6.8)	21246 (5)	0.078	4234 (6)	28903 (4.7)	0.058
**Antidiabetic drugs use‡**									
Insulin	1975 (6.6)	13651 (7.4)	-.033	3811 (9.5)	46039 (10.8)	-.043	5786 (8.2)	59690 (9.8)	-.054

© 2023 Bea S et al. *JAMA Network Open.*

α-glucosidase inhibitors	546 (1.8)	5022 (2.7)	-.061	1465 (3.6)	21874 (5.1)	-.072	2011 (2.9)	26896 (4.4)	-.082
GLP-1RA	114 (0.4)	139 (0.1)	0.064	106 (0.3)	271 (0.1)	0.050	220 (0.3)	410 (0.1)	0.056
Meglitinides	80 (0.3)	764 (0.4)	-.025	241 (0.6)	3654 (0.9)	-.030	321 (0.5)	4418 (0.7)	-.035
Metformin	15080 (50.2)	99732 (54.1)	-.078	23675 (58.9)	268303 (62.9)	-.082	38755 (55.2)	368035 (60.2)	-.103
Sulfonylureas	6094 (20.3)	50807 (27.6)	-.171	10762 (26.8)	146399 (34.3)	-.164	16856 (24)	197206 (32.3)	-.185
Thiazolidinediones	1691 (5.6)	8056 (4.4)	0.058	3102 (7.7)	24514 (5.7)	0.079	4793 (6.8)	32570 (5.3)	0.063
Level of antidiabetic treatments§			0.136			0.116			0.149
1	22603 (75.2)	128019 (69.4)		26712 (66.5)	258474 (60.6)		49315 (70.2)	386493 (63.3)	
2	5482 (18.2)	42723 (23.2)		9658 (24)	121980 (28.6)		15140 (21.6)	164703 (27)	
3	1975 (6.6)	13651 (7.4)		3811 (9.5)	46039 (10.8)		5786 (8.2)	59690 (9.8)	
**Diabetic complications‡**									
Retinopathy	792 (2.6)	5080 (2.8)	-.007	1279 (3.2)	15054 (3.5)	-.019	2071 (2.9)	20134 (3.3)	-.020
Neuropathy	2415 (8)	16834 (9.1)	-.039	4545 (11.3)	55158 (12.9)	-.050	6960 (9.9)	71992 (11.8)	-.060
Nephropathy	3009 (10)	19434 (10.5)	-.017	6359 (15.8)	73185 (17.2)	-.036	9368 (13.3)	92619 (15.2)	-.052
**CCI groups‡**			0.096			0.137			0.152
0	9596 (31.9)	50996 (27.7)		9919 (24.7)	86175 (20.2)		19515 (27.8)	137171 (22.5)	
1-2	13613 (45.3)	85478 (46.4)		18209 (45.3)	193789 (45.4)		31822 (45.3)	279267 (45.7)	
≥3	6851 (22.8)	47919 (26)		12053 (30)	146529 (34.4)		18904 (26.9)	194448 (31.8)	
**Health examination data (mean, SD)**									
BMI, kg/m^2	29.7 (3.8)	28.4 (3.5)		25.1 (2.7)	24.2 (2.7)		27.1 (3.9)	25.5 (3.5)	
WC, cm	96.2 (9.9)	94.2 (8.1)		84.3 (6.9)	83.3 (7.1)		89.4 (10.2)	86.6 (9.0)	
Total cholesterol, mg/dL	204.0 (52.0)	203.8 (53.6)		185.9 (46.5)	185.3 (45.6)		193.6 (49.7)	190.9 (48.9)	
HDL cholesterol, mg/dL	48.3 (13.8)	48.0 (15.9)		52.5 (14.5)	51.8 (23.2)		50.7 (14.4)	50.7 (21.4)	
LDL cholesterol, mg/dL	110.4 (47.0)	108.7 (46.9)		106.9 (42.9)	106.7 (41.6)		108.4 (44.8)	107.3 (43.2)	
TG, mg/dL	255.1 (205.6)	265.1 (205.8)		133.8 (70.4)	135.4 (72.3)		185.7 (156.6)	174.5 (141.3)	
Scr, mg/dL	0.9 (0.4)	0.9 (0.6)		0.9 (0.7)	0.9 (0.6)		0.9 (0.6)	0.9 (0.6)	
AST, IU/l	39.3 (32.1)	38.8 (34.4)		27.1 (15.8)	26.4 (17.3)		32.3 (24.9)	30.2 (24.4)	
ALT, IU/l	48.0 (37.3)	45.1 (39.3)		28.9 (20.2)	26.9 (20.3)		37.1 (30.3)	32.4 (28.7)	
GGT, U/l	83.8 (87.5)	94.9 (108.9)		33.9 (28.3)	34.5 (34.4)		55.2 (65.9)	52.7 (71.9)	
GFR, ml/min per 1.73m^2	90.6 (27.2)	87.5 (27.5)		88.8 (27.2)	85.6 (27.1)		89.6 (27.2)	86.2 (27.3)	
FBG, mg/dL	152.0 (51.6)	156.5 (55.0)		141.1 (48.9)	144.8 (52.5)		145.8 (50.4)	148.3 (53.6)	

Abbreviation: ALT, serum alanine transaminase; AST, serum aspartate transaminase; BMI, body mass index; CCB, calcium channel blocker; CCI, Charlson's comorbidity index; COPD, chronic obstructive pulmonary disease; DPP-4i, dipeptidyl peptidase 4 inhibitors; FBG, fasting blood glucose level; GFR, glomerular filtration rate; GGT, γ-glutamyl transferase; HDL, high-density cholesterol; LDL, low-density cholesterol; NAFLD, non-alcoholic fatty liver disease; NSAID, nonsteroidal anti-inflammatory drugs; WC, waist circumference; Scr, serum creatinine; SMD, standardized mean difference; SGLT-2i, sodium glucose cotransporter 2 inhibitors; TG, total glyceride; RAS, renin-angiotensin system

§Use of antidiabetic drugs previous 365 days before the entry date of cohort: level 1, Patients not received any of antidiabetics or received only one antidiabetics; level 2, Patients received ≥2 different classes of antidiabetics without insulin; level 3, Patients received ≥1 insulin either unaccompanied or in combination with other antidiabetics.

‡Assessed in the years before study cohort entry.

© 2023 Bea S et al. *JAMA Network Open*.

eTable 7. Baseline Characteristics Before 1:1 Propensity Score Matching: Patients Initiating GLP-1RAs vs DPP-4 Inhibitors Overall and Across NAFLD Status

	With NAFLD			Without NAFLD			Overall Population		
	**GLP-1RA**	**DPP-4i**	**SMD**	**GLP-1RA**	**DPP-4i**	**SMD**	**GLP-1RA**	**DPP-4i**	**SMD**
**Number of patients**	7291	227545		13045	533523		20336	761068	
**Fatty liver index, mean (SD)**	79.5 (11.4)	76.9 (10.6)		30.2 (16.4)	30.0 (16.0)		48.3 (28.0)	43.7 (25.9)	
**Age, years; mean (SD)**	56.0 (10.2)	58.2 (10.7)	-.211	60.6 (10.0)	63.6 (10.7)	-.291	58.9 (10.3)	62.0 (11.0)	-.286
**Male, No. (%)**	4097 (56.2)	159973 (70.3)	0.296	6114 (46.9)	273565 (51.3)	0.088	10211 (50.2)	433538 (57)	0.136
**Calendar year**			1.202			1.535			1.460
2013	66 (0.9)	27362 (12)		36 (0.3)	74426 (13.9)		102 (0.5)	101788 (13.4)	
2014	26 (0.4)	26798 (11.8)		20 (0.2)	69636 (13.1)		46 (0.2)	96434 (12.7)	
2015	82 (1.1)	30139 (13.2)		59 (0.5)	76992 (14.4)		141 (0.7)	107131 (14.1)	
2016	550 (7.5)	32712 (14.4)		839 (6.4)	77762 (14.6)		1389 (6.8)	110474 (14.5)	
2017	1474 (20.2)	30939 (13.6)		2684 (20.6)	69438 (13)		4158 (20.4)	100377 (13.2)	
2018	1705 (23.4)	28941 (12.7)		3267 (25)	60642 (11.4)		4972 (24.4)	89583 (11.8)	
2019	1952 (26.8)	26177 (11.5)		3455 (26.5)	54782 (10.3)		5407 (26.6)	80959 (10.6)	
2020	1436 (19.7)	24477 (10.8)		2685 (20.6)	49845 (9.3)		4121 (20.3)	74322 (9.8)	
**Healthcare use**‡									
Inpatient hospitalizations			0.272			0.264			0.289
0	5195 (71.3)	183169 (80.5)		9191 (70.5)	415000 (77.8)		14386 (70.7)	598169 (78.6)	
1-2	1788 (24.5)	39442 (17.3)		3363 (25.8)	104183 (19.5)		5151 (25.3)	143625 (18.9)	
≥3	308 (4.2)	4934 (2.2)		491 (3.8)	14340 (2.7)		799 (3.9)	19274 (2.5)	
Number of physician visits			0.644			0.784			0.728
0-2	53 (0.7)	16839 (7.4)		55 (0.4)	30865 (5.8)		108 (0.5)	47704 (6.3)	
3-5	173 (2.4)	20596 (9.1)		183 (1.4)	35778 (6.7)		356 (1.8)	56374 (7.4)	
≥6	7065 (96.9)	190110 (83.5)		12807 (98.2)	466880 (87.5)		19872 (97.7)	656990 (86.3)	
**Comorbidities**‡									
Dyslipidemia	3648 (50)	83239 (36.6)	0.365	6899 (52.9)	192868 (36.1)	0.492	10547 (51.9)	276107 (36.3)	0.448
Hypertension	4068 (55.8)	126834 (55.7)	0.144	5743 (44)	260577 (48.8)	0.094	9811 (48.2)	387411 (50.9)	0.124
Atrial fibrillation	107 (1.5)	2979 (1.3)	0.029	195 (1.5)	7875 (1.5)	0.026	302 (1.5)	10854 (1.4)	0.027
Heart failure	41 (0.6)	842 (0.4)	0.036	55 (0.4)	1483 (0.3)	0.035	96 (0.5)	2325 (0.3)	0.036
Liver cirrhosis	318 (4.4)	2645 (1.2)	0.204	469 (3.6)	7206 (1.4)	0.165	787 (3.9)	9851 (1.3)	0.179
Chronic kidney disease	43 (0.6)	1461 (0.6)	0.004	126 (1)	6527 (1.2)	-.002	169 (0.8)	7988 (1)	-.004
Dementia	433 (5.9)	8959 (3.9)	0.117	774 (5.9)	25476 (4.8)	0.097	1207 (5.9)	34435 (4.5)	0.102
Depression	240 (3.3)	3841 (1.7)	0.118	421 (3.2)	12744 (2.4)	0.082	661 (3.3)	16585 (2.2)	0.092
Hypothyroidism	55 (0.8)	1308 (0.6)	0.032	130 (1)	5105 (1)	0.024	185 (0.9)	6413 (0.8)	0.024
Hyperthyrodism	156 (2.1)	3553 (1.6)	0.059	245 (1.9)	8297 (1.6)	0.050	401 (2)	11850 (1.6)	0.054
Gallbladder disease	401 (5.5)	12042 (5.3)	0.041	739 (5.7)	34190 (6.4)	0.022	1140 (5.6)	46232 (6.1)	0.027
COPD	554 (7.6)	13156 (5.8)	0.104	1206 (9.2)	38668 (7.2)	0.128	1760 (8.7)	51824 (6.8)	0.117
**Comedication**‡									
Acetaminophen	4871 (66.8)	131569 (57.8)	0.326	8587 (65.8)	326159 (61.1)	0.335	13458 (66.2)	457728 (60.1)	0.331
RAS inhibitors	5056 (69.3)	117270 (51.5)	0.488	6874 (52.7)	228461 (42.8)	0.369	11930 (58.7)	345731 (45.4)	0.424
CCB	3365 (46.2)	95937 (42.2)	0.189	4274 (32.8)	187810 (35.2)	0.095	7639 (37.6)	283747 (37.3)	0.142
β-blockers	1645 (22.6)	38707 (17)	0.197	2285 (17.5)	78998 (14.8)	0.157	3930 (19.3)	117705 (15.5)	0.177
Diuretics	2278 (31.2)	63499 (27.9)	0.154	2830 (21.7)	126739 (23.8)	0.063	5108 (25.1)	190238 (25)	0.106
Systemic antibiotics	5219 (71.6)	143181 (62.9)	0.338	9265 (71)	347014 (65)	0.384	14484 (71.2)	490195 (64.4)	0.368
Oral anticoagulants	150 (2.1)	3144 (1.4)	0.067	258 (2)	8828 (1.7)	0.050	408 (2)	11972 (1.6)	0.055
Oral antiplatelets	3003 (41.2)	63717 (28)	0.354	6008 (46.1)	174163 (32.6)	0.416	9011 (44.3)	237880 (31.3)	0.388
NSAIDs	4908 (67.3)	134462 (59.1)	0.315	8816 (67.6)	333210 (62.5)	0.351	13724 (67.5)	467672 (61.4)	0.336
Opioids	983 (13.5)	21848 (9.6)	0.162	1656 (12.7)	56086 (10.5)	0.137	2639 (13)	77934 (10.2)	0.145
Systemic corticosteroids	3689 (50.6)	108034 (47.5)	0.183	6831 (52.4)	270878 (50.8)	0.229	10520 (51.7)	378912 (49.8)	0.210
Statins	5820 (79.8)	104954 (46.1)	0.833	10555 (80.9)	259889 (48.7)	0.921	16375 (80.5)	364843 (47.9)	0.887
Other lipid-lowering agents	2459 (33.7)	34391 (15.1)	0.487	3249 (24.9)	54489 (10.2)	0.456	5708 (28.1)	88880 (11.7)	0.477
Vitamin E	1000 (13.7)	15359 (6.7)	0.260	1158 (8.9)	25828 (4.8)	0.203	2158 (10.6)	41187 (5.4)	0.231
Nitrates	456 (6.3)	9498 (4.2)	0.119	942 (7.2)	25863 (4.8)	0.144	1398 (6.9)	35361 (4.6)	0.134
**Antidiabetic drugs use**‡									
Insulin	2964 (40.7)	15322 (6.7)	0.872	6407 (49.1)	51903 (9.7)	1.016	9371 (46.1)	67225 (8.8)	0.955
α-glucosidase inhibitors	168 (2.3)	8101 (3.6)	-.047	418 (3.2)	33698 (6.3)	-.094	586 (2.9)	41799 (5.5)	-.086

© 2023 Bea S et al. *JAMA Network Open.*

Meglitinides	64 (0.9)	1059 (0.5)	0.058	195 (1.5)	4886 (0.9)	0.072	259 (1.3)	5945 (0.8)	0.064
Metformin	6520 (89.4)	128584 (56.5)	0.893	11722 (89.9)	342986 (64.3)	0.897	18242 (89.7)	471570 (62)	0.887
Sulfonylureas	4911 (67.4)	70153 (30.8)	0.847	8817 (67.6)	202044 (37.9)	0.787	13728 (67.5)	272197 (35.8)	0.799
Thiazolidinediones	1400 (19.2)	9995 (4.4)	0.483	2735 (21)	30585 (5.7)	0.501	4135 (20.3)	40580 (5.3)	0.491
Level of antidiabetic treatments§			1.742			1.875			1.818
1	292 (4)	148884 (65.4)		273 (2.1)	304510 (57.1)		565 (2.8)	453394 (59.6)	
2	4035 (55.3)	63339 (27.8)		6365 (48.8)	177110 (33.2)		10400 (51.1)	240449 (31.6)	
3	2964 (40.7)	15322 (6.7)		6407 (49.1)	51903 (9.7)		9371 (46.1)	67225 (8.8)	
**Diabetic complications‡**									
Retinopathy	865 (11.9)	6287 (2.8)	0.365	1417 (10.9)	18436 (3.5)	0.322	2282 (11.2)	24723 (3.2)	0.336
Neuropathy	1533 (21)	21065 (9.3)	0.365	3399 (26.1)	68904 (12.9)	0.411	4932 (24.3)	89969 (11.8)	0.387
Nephropathy	2164 (29.7)	22892 (10.1)	0.535	5164 (39.6)	86646 (16.2)	0.625	7328 (36)	109538 (14.4)	0.580
**CCI groups‡**			0.685			0.700			0.699
0	844 (11.6)	61426 (27)		1376 (10.5)	108630 (20.4)		2220 (10.9)	170056 (22.3)	
1-2	2781 (38.1)	106727 (46.9)		4500 (34.5)	244501 (45.8)		7281 (35.8)	351228 (46.1)	
≥3	3666 (50.3)	59392 (26.1)		7169 (55)	180392 (33.8)		10835 (53.3)	239784 (31.5)	
**Health examination data (mean, SD)**									
BMI, kg/m^2	3.5 (4.2)	28.3 (3.4)		24.7 (2.9)	24.2 (27.3)		26.8 (4.4)	25.4 (3.5)	
WC, cm	98.6 (9.3)	94.0 (8.2)		84.3 (7.5)	83.2 (7.1)		89.6 (10.7)	86.3 (8.9)	
Total cholesterol, mg/dL	179.6 (48.9)	203.7 (53.0)		163.3 (42.6)	185.9 (45.0)		169.2 (45.7)	191.1 (48.2)	
HDL cholesterol, mg/dL	46.8 (14.4)	48.2 (18.9)		50.2 (12.9)	51.5 (25.3)		49.0 (13.6)	50.6 (23.7)	
LDL cholesterol, mg/dL	90.0 (42.7)	108.4 (74.8)		87.2 (37.8)	107.4 (44.2)		88.2 (39.7)	107.7 (54.7)	
TG, mg/dL	238.4 (200.8)	267.0 (207.0)		130.4 (69.6)	136.9 (73.2)		170.0 (143.3)	174.7 (140.6)	
Scr, mg/dL	0.9 (0.5)	1.0 (0.7)		0.9 (0.4)	0.9 (0.7)		0.9 (0.4)	0.9 (0.7)	
AST, IU/l	36.9 (26.5)	38.3 (35.8)		25.8 (15.8)	26.1 (16.7)		29.9 (21.1)	29.6 (24.6)	
ALT, IU/l	42.2 (30.3)	44.6 (42.0)		26.8 (18.2)	26.7 (20.6)		32.5 (24.5)	31.9 (29.6)	
GGT, U/l	69.4 (75.6)	94.5 (109.4)		29.8 (30.1)	34.1 (34.1)		44.3 (55.1)	51.7 (71.1)	
GFR, ml/min per 1.73m^2	88.7 (32.2)	87.5 (28.1)		86.5 (26.8)	84.3 (27.8)		87.3 (28.9)	85.2 (27.9)	
FBG, mg/dL	167.9 (62.9)	156.5 (55.0)		158.1 (62.0)	145.1 (52.6)		161.7 (35.0)	148.5 (53.6)	

Abbreviation: ALT, serum alanine transaminase; AST, serum aspartate transaminase; BMI, body mass index; CCB, calcium channel blocker; CCI, Charlson's comorbidity index; COPD, chronic obstructive pulmonary disease; DPP-4i, dipeptidyl peptidase 4 inhibitors; FBG, fasting blood glucose level; GFR, glomerular filtration rate; GGT, γ-glutamyl transferase; GLP-1RA, glucagon-like peptide-1 receptor agonists; HDL, high-density cholesterol; LDL, low-density cholesterol; NAFLD, non-alcoholic fatty liver disease; NSAID, nonsteroidal anti-inflammatory drugs; WC, waist circumference; Scr, serum creatinine; SMD, standardized mean difference; TG, total glyceride; RAS, renin-angiotensin system

§Use of antidiabetic drugs previous 365 days before the entry date of cohort: level 1, Patients not received any of antidiabetics or received only one antidiabetics; level 2, Patients received ≥2 different classes of antidiabetics without insulin; level 3, Patients received ≥1 insulin either unaccompanied or in combination with other antidiabetics.

‡Assessed in the years before study cohort entry.

© 2023 Bea S et al. *JAMA Network Open.*

eTable 8. Sensitivity Analyses of Effectiveness Outcomes for the 1:1 Propensity Score–Matched Cohort of New Users of Sodium-Glucose Cotransporter-2 Inhibitors (SGLT-2i) or Glucagon-Like Peptide-1 Receptor Agonists (GLP-1RA) and New Users of Dipeptidyl Peptidase-4 Inhibitors (DPP-4i), by Non-Alcoholic Fatty Liver Disease (NAFLD) Status: Intention-to-Treat Analysis

	**Events, n (Incidence Rate per 1000 Person-Years)**		**Rate Difference per 1000 Person-Years (95% CI)**	**Hazard Ratio (95% CI)**	**P value for Homogeneity**
**SGLT-2i vs DPP-4i**					
**MACE**					
High risk NAFLD	252 (5.74)	370 (7.74)	-2.00 (-3.07 to -0.94)	0.73 (0.62 to 0.86)	0.3110
Without risk NAFLD	472 (8.22)	687 (9.60)	-1.38 (-2.41 to -0.35)	0.81 (0.72 to 0.91)	
Overall population	724 (7.15)	1057 (8.86)	-0.82 (-1.19 to -0.46)	0.78 (0.71 to 0.85)	
**HHF**					
With NAFLD	34 (0.77)	49 (1.02)	-0.25 (-0.63 to 0.14)	0.76 (0.49 to 1.17)	0.2752
Without NAFLD	51 (0.88)	107 (1.48)	-0.60 (-0.97 to -0.23)	0.56 (0.40 to 0.78)	
Overall population	85 (0.84)	156 (1.30)	-0.46 (-0.73 to -0.19)	0.62 (0.48 to 0.81)	
**GLP-1RA vs DPP-4i**					
**MACE**					
With NAFLD	46 (8.29)	78 (14.15)	-5.86 (-9.81 to -1.92)	0.59 (0.41 to 0.84)	0.7487
Without NAFLD	108 (10.89)	198 (19.96)	-9.08 (-12.5 to -5.62)	0.55 (0.43 to 0.69)	
Overall population	154 (9.95)	276 (17.89)	-7.93 (-10.6 to -5.30)	0.56 (0.46 to 0.68)	
**HHF**					
With NAFLD	19 (3.42)	18 (3.25)	0.17 (-1.98 to 2.31)	1.05 (0.55 to 2.00)	0.1170
Without NAFLD	19 (1.91)	36 (3.60)	-1.69 (-3.15 to -0.24)	0.53 (0.30 to 0.92)	
Overall population	38 (2.45)	54 (3.47)	-1.03 (-2.24 to 0.18)	0.70 (0.47 to 1.07)	

Abbreviation: CI, confidence interval; DPP-4i, dipeptidyl peptidase 4 inhibitors; GLP-1RA, glucagon-like peptide-1 receptor agonists; HHF, hospitalization for heart failure; MACE, major adverse cardiovascular events; NAFLD, non-alcoholic fatty liver disease; SGLT-2i, sodium glucose cotransporter 2 inhibitors

© 2023 Bea S et al. *JAMA Network Open.*

eTable 9. Sensitivity Analyses of Effectiveness Outcomes for the 1:1 Propensity Score–Matched Cohort of New Users of Sodium-Glucose Cotransporter-2 Inhibitors (SGLT-2i) or Glucagon-Like Peptide-1 Receptor Agonists (GLP-1RA) and New Users of Dipeptidyl Peptidase-4 Inhibitors (DPP-4i), by Non-Alcoholic Fatty Liver Disease (NAFLD) Status: Varying Grace Period to 90 Days

	**Events, n (Incidence Rate per 1000 Person-Years)**		**Rate Difference per 1000 Person-Years (95% CI)**	**Hazard Ratio (95% CI)**	**P value for Homogeneity**
	**Drugs of interests**	**Comparator**			
**SGLT-2i vs DPP-4i**					
**MACE**					
With NAFLD	264 (5.73)	397 (7.79)	-2.07 (-3.10 to -1.03)	0.72 (0.62 to 0.85)	0.2322
Without NAFLD	495 (8.23)	733 (9.66)	-1.43 (-2.44 to -0.42)	0.81 (0.72 to 0.90)	
Overall population	759 (7.15)	1130 (8.91)	-1.77 (-2.49 to -1.04)	0.77 (0.70 to 0.85)	
**HHF**					
With NAFLD	37 (0.80)	58 (1.13)	-0.33 (-0.72 to 0.06)	0.71 (0.47 to 1.07)	0.4481
Without NAFLD	55 (0.91)	114 (1.49)	-0.58 (-0.94 to -0.21)	0.58 (0.42 to 0.80)	
Overall population	92 (0.86)	172 (1.34)	-0.48 (-0.75 to -0.22)	0.62 (0.48 to 0.80)	
**GLP-1RA vs DPP-4i**					
**MACE**					
With NAFLD	42 (8.28)	121 (11.83)	-3.55 (-6.82 to -0.27)	0.63 (0.44 to 0.91)	0.3479
Without NAFLD	83 (9.57)	275 (14.86)	-5.29 (-7.99 to -2.58)	0.51 (0.40 to 0.66)	
Overall population	125 (9.10)	396 (13.78)	-4.68 (-6.78 to -2.59)	0.54 (0.44 to 0.67)	
**HHF**					
With NAFLD	12 (2.36)	27 (2.61)	-0.25 (-1.91 to 1.41)	0.80 (0.40 to 1.62)	0.2392
Without NAFLD	14 (1.61)	55 (2.94)	-1.33 (-2.48 to -0.18)	0.46 (0.25 to 0.83)	
Overall population	26 (1.89)	82 (2.82)	-0.94 (-1.88 to 0.01)	0.57 (0.36 to 0.90)	

Abbreviation: CI, confidence interval; DPP-4i, dipeptidyl peptidase 4 inhibitors; GLP-1RA, glucagon-like peptide-1 receptor agonists; HHF, hospitalization for heart failure; MACE, major adverse cardiovascular events; NAFLD, non-alcoholic fatty liver disease; SGLT-2i, sodium glucose cotransporter 2 inhibitors

© 2023 Bea S et al. *JAMA Network Open.*

eTable 10. Sensitivity Analyses of Effectiveness Outcomes for the 1:1 Propensity Score–Matched Cohort of New Users of Sodium-Glucose Cotransporter-2 Inhibitors (SGLT-2i) or Glucagon-Like Peptide-1 Receptor Agonists (GLP-1RA) and New Users of Dipeptidyl Peptidase-4 Inhibitors (DPP-4i), by Non-Alcoholic Fatty Liver Disease (NAFLD) Status: Varying Grace Period to 45 Days

	Events, n (Incidence Rate per 1000 Person-Years)		Rate Difference per 1000 Person-Years (95% CI)	Hazard Ratio (95% CI)	P value for Homogeneity
	Drugs of interests	Comparator			
**SGLT-2i vs DPP-4i**					
**MACE**					
With NAFLD	243 (5.76)	352 (7.78)	-2.01 (-3.10 to -0.93)	0.73 (0.62 to 0.86)	0.2164
Without NAFLD	458 (8.30)	647 (9.50)	-1.19 (-2.25 to -0.14)	0.83 (0.73 to 0.93)	
Overall population	701 (7.20)	999 (8.81)	-1.61 (-2.37 to -0.84)	0.79 (0.71 to 0.87)	
**HHF**					
With NAFLD	34 (0.80)	49 (1.08)	-0.27 (-0.68 to 0.13)	0.75 (0.48 to 1.16)	0.3692
Without NAFLD	49 (0.88)	98 (1.43)	-0.54 (-0.92 to -0.17)	0.58 (0.41 to 0.82)	
Overall population	83 (0.85)	147 (1.29)	-0.44 (-0.71 to -0.16)	0.64 (0.49 to 0.84)	
**GLP-1RA vs DPP-4i**					
**MACE**					
With NAFLD	20 (7.20)	104 (11.44)	-4.24 (-8.08 to -0.39)	0.47 (0.28 to 0.77)	0.9436
Without NAFLD	51 (10.88)	243 (14.42)	-3.54 (-7.03 to -0.05)	0.46 (0.33 to 0.62)	
Overall population	71 (9.51)	347 (13.37)	-3.86 (-6.49 to -1.24)	0.46 (0.35 to 0.60)	
**HHF**					
With NAFLD	9 (3.24)	24 (2.62)	0.62 (-1.74 to 2.98)	0.99 (0.44 to 2.22)	0.1981
Without NAFLD	8 (1.70)	44 (2.59)	-0.88 (-2.29 to 0.52)	0.47 (0.21 to 1.03)	
Overall population	17 (2.27)	68 (2.60)	-0.32 (-1.57 to 0.92)	0.65 (0.37 to 1.14)	

Abbreviation: CI, confidence interval; DPP-4i, dipeptidyl peptidase 4 inhibitors; GLP-1RA, glucagon-like peptide-1 receptor agonists; HHF, hospitalization for heart failure; MACE, major adverse cardiovascular events; NAFLD, non-alcoholic fatty liver disease; SGLT-2i, sodium glucose cotransporter 2 inhibitors

© 2023 Bea S et al. *JAMA Network Open.*

eTable 11. Sensitivity Analyses of Effectiveness Outcomes for the 1:1 Propensity Score–Matched Cohort of New Users of Sodium-Glucose Cotransporter-2 Inhibitors (SGLT-2i) or Glucagon-Like Peptide-1 Receptor Agonists (GLP-1RA) and New Users of Dipeptidyl Peptidase-4 Inhibitors (DPP-4i), by Non-Alcoholic Fatty Liver Disease (NAFLD) Status: Restricted Cohort Within 1 Year of Fatty Liver Index

	Events, n (Incidence Rate per 1000 Person-Years)		Rate Difference per 1000 Person-Years (95% CI)	Hazard Ratio (95% CI)	P value for Homogeneity
	Drugs of interests	Comparator			
**SGLT-2i vs DPP-4i**					
**MACE**					
With NAFLD	176 (5.36)	266 (7.31)	-1.95 (-3.13 to -0.76)	0.73 (0.60 to 0.88)	0.7417
Without NAFLD	295 (6.75)	469 (8.57)	-1.82 (-2.92 to -0.73)	0.76 (0.66 to 0.88)	
Overall population	471 (6.15)	735 (8.07)	-1.91 (-2.72 to -1.11)	0.75 (0.66 to 0.84)	
**HHF**					
With NAFLD	20 (0.61)	38 (1.04)	-0.43 (-0.85 to -0.01)	0.58 (0.34 to 1.00)	0.5947
Without NAFLD	29 (0.66)	73 (1.32)	-0.66 (-1.05 to -0.27)	0.48 (0.31 to 0.75)	
Overall population	49 (0.64)	111 (1.21)	-0.57 (-0.86 to -0.28)	0.52 (0.37 to 0.72)	
**GLP-1RA vs DPP-4i**					
**MACE**					
With NAFLD	16 (6.58)	69 (10.11)	-3.52 (-7.53 to 0.49)	0.53 (0.30 to 0.93)	0.5825
Without NAFLD	41 (9.69)	145 (11.68)	-1.99 (-5.52 to 1.53)	0.64 (0.44 to 0.91)	
Overall population	57 (8.56)	214 (11.12)	-2.57 (-5.24 to 0.11)	0.60 (0.44 to 0.81)	
**HHF**					
With NAFLD	7 (2.88)	23 (3.35)	-0.47 (-3.01 to 2.06)	0.73 (0.30 to 1.76)	0.7904
Without NAFLD	5 (1.18)	24 (1.91)	-0.73 (-2.02 to 0.55)	0.61 (0.23 to 1.65)	
Overall population	12 (1.80)	47 (2.42)	-0.62 (-1.85 to 0.61)	0.67 (0.35 to 1.30)	

Abbreviation: CI, confidence interval; DPP-4i, dipeptidyl peptidase 4 inhibitors; GLP-1RA, glucagon-like peptide-1 receptor agonists; HHF, hospitalization for heart failure; MACE, major adverse cardiovascular events; NAFLD, non-alcoholic fatty liver disease; SGLT-2i, sodium glucose cotransporter 2 inhibitors

© 2023 Bea S et al. *JAMA Network Open.*

eTable 12. Sensitivity Analyses of Effectiveness Outcomes for the 1:1 Propensity Score–Matched Cohort of New Users of Sodium-Glucose Cotransporter-2 Inhibitors (SGLT-2i) or Glucagon-Like Peptide-1 Receptor Agonists (GLP-1RA) and New Users of Dipeptidyl Peptidase-4 Inhibitors (DPP-4i), by Non-Alcoholic Fatty Liver Disease (NAFLD) Status: Using Alternative NAFLD Definition; Hepatic Steatosis Index

	**Events, n (Incidence Rate per 1000 Person-Years)**		**Rate Difference per 1000 Person-Years (95% CI)**	**Hazard Ratio (95% CI)**	**P value for Homogeneity**
	**Drugs of interests**	**Comparator**			
**SGLT-2i vs DPP-4i**					
**MACE**					
With NAFLD	398 (5.43)	596 (7.21)	-1.77 (-2.56 to -0.99)	0.76 (0.67 to 0.87)	0.1942
Without NAFLD	326 (11.63)	465 (12.82)	-1.19 (-2.91 to 0.53)	0.87 (0.76 to 1.01)	
Overall population	724 (7.15)	1,061 (8.92)	-1.77 (-2.52 to -1.02)	0.81 (0.74 to 0.89)	
**HHF**					
With NAFLD	45 (0.61)	85 (1.02)	-0.41 (-0.69 to -0.13)	0.69 (0.48 to 1.00)	0.8377
Without NAFLD	40 (1.42)	72 (1.96)	-0.55 (-1.18 to 0.09)	0.73 (0.49 to 1.08)	
Overall population	85 (0.84)	157 (1.31)	-0.47 (-0.74 to -0.20)	0.72 (0.55 to 0.94)	
**GLP-1RA vs DPP-4i**					
**MACE**					
With NAFLD	32 (6.51)	174 (10.03)	-3.52 (-5.99 to -1.05)	0.53 (0.37 to 0.75)	1.0000
Without NAFLD	43 (12.60)	175 (17.83)	-5.23 (-9.83 to -0.63)	0.53 (0.38 to 0.76)	
Overall population	85 (8.62)	349 (12.85)	-4.23 (-6.51 to -1.96)	0.53 (0.41 to 0.68)	
**HHF**					
With NAFLD	14 (2.17)	32 (1.83)	0.34 (-0.96 to 1.64)	1.45 (0.71 to 2.96)	0.0273
Without NAFLD	6 (1.75)	32 (3.21)	-1.46 (-3.25 to 0.33)	0.36 (0.13 to 0.98)	
Overall population	20 (2.02)	64 (2.33)	-0.31 (-1.36 to 0.75)	0.90 (0.52 to 1.55)	

Abbreviation: CI, confidence interval; DPP-4i, dipeptidyl peptidase 4 inhibitors; GLP-1RA, glucagon-like peptide-1 receptor agonists; HHF, hospitalization for heart failure; MACE, major adverse cardiovascular events; NAFLD, non-alcoholic fatty liver disease; SGLT-2i, sodium glucose cotransporter 2 inhibitors

© 2023 Bea S et al. *JAMA Network Open.*

eTable 13. Sensitivity Analyses of Effectiveness Outcomes for the 1:1 Propensity Score–Matched Cohort of New Users of Sodium-Glucose Cotransporter-2 Inhibitors (SGLT-2i) or Glucagon-Like Peptide-1 Receptor Agonists (GLP-1RA) and New Users of Dipeptidyl Peptidase-4 Inhibitors (DPP-4i), by Non-Alcoholic Fatty Liver Disease (NAFLD) Status: Propensity Score Based Fine Stratification Within Average Treatment Estimate Among Whole Population

	**Events, n (Incidence Rate per 1000 Person-Years)**		**Rate Difference per 1000 Person-Years (95% CI)**	**Hazard Ratio (95% CI)**	**P value for Homogeneity**
	**Drugs of interests**	**Comparator**			
**SGLT-2i vs DPP-4i**					
**MACE**					
With NAFLD	252 (5.73)	3,649 (9.99)	-4.26 (-5.05 to -3.47)	0.71 (0.61 to 0.83)	0.2108
Without NAFLD	472 (8.22)	10,994 (12.09)	-3.87 (-4.65 to -3.09)	0.80 (0.72 to 0.89)	
Overall population	724 (7.14)	14,643 (11.49)	-4.35 (-5.07 to -3.63)	0.77 (0.70 to 0.84)	
**HHF**					
With NAFLD	34 (0.77)	613 (1.66)	-0.89 (-1.20 to -0.58)	0.53 (0.35 to 0.79)	0.8869
Without NAFLD	51 (0.88)	1,926 (2.09)	-1.21 (-1.48 to -0.94)	0.51 (0.36 to 0.71)	
Overall population	85 (0.83)	2,539 (1.97)	-1.14 (-1.34 to -0.95)	0.52 (0.40 to 0.67)	
**GLP-1RA vs DPP-4i**					
**MACE**					
With NAFLD	34 (8.11)	5,101 (9.73)	-1.62 (-4.49 to 1.25)	0.35 (0.14 to 0.91)	0.5456
Without NAFLD	70 (9.85)	15,621 (11.75)	-1.90 (-4.29 to 0.49)	0.25 (0.14 to 0.43)	
Overall population	104 (9.21)	20,722 (11.18)	-1.97 (-3.79 to -0.15)	0.46 (0.19 to 1.12)	
**HHF**					
With NAFLD	11 (2.62)	880 (1.66)	0.96 (-0.73 to 2.65)	0.39 (0.22 to 0.48)	0.1465
Without NAFLD	11 (1.54)	2,730 (2.03)	-0.49 (-1.49 to 0.51)	0.41 (0.10 to 0.72)	
Overall population	22 (1.94)	3,610 (1.92)	0.02 (-0.84 to 0.88)	0.22 (0.11 to 0.44)	

Abbreviation: CI, confidence interval; DPP-4i, dipeptidyl peptidase 4 inhibitors; GLP-1RA, glucagon-like peptide-1 receptor agonists; HHF, hospitalization for heart failure; MACE, major adverse cardiovascular events; NAFLD, non-alcoholic fatty liver disease; SGLT-2i, sodium glucose cotransporter 2 inhibitors

© 2023 Bea S et al. *JAMA Network Open.*

eTable 14. Sensitivity Analyses of Effectiveness Outcomes for the 1:1 Propensity Score–Matched Cohort of New Users of Sodium-Glucose Cotransporter-2 Inhibitors (SGLT-2i) or Glucagon-Like Peptide-1 Receptor Agonists (GLP-1RA) and New Users of Dipeptidyl Peptidase-4 Inhibitors (DPP-4i), by Non-Alcoholic Fatty Liver Disease (NAFLD) Status: Treating All-Cause Death as Competing Event

	Events, n (Incidence Rate per 1000 Person-Years)		Rate Difference per 1000 Person-Years (95% CI)	Hazard Ratio (95% CI)	P value for Homogeneity
	Drugs of interests	Comparator			
**SGLT-2i vs DPP-4i**					
**MACE**					
With NAFLD	252 (9.29)	370 (16.53)	-7.24 (-8.71 to -5.78)	0.75 (0.64 to 0.88)	0.2234
Without NAFLD	472 (14.53)	687 (22.83)	-8.30 (-9.78 to -6.81)	0.85 (0.75 to 0.96)	
Overall population	724 (12.26)	1057 (20.31)	-8.05 (-9.10 to -6.99)	0.81 (0.74 to 0.89)	
**HHF**					
With NAFLD	34 (5.40)	49 (11.82)	-6.43 (-7.62 to -5.24)	0.84 (0.55 to 1.30)	0.3222
Without NAFLD	51 (9.24)	107 (18.01)	-8.77 (-10.0 to -7.52)	0.64 (0.45 to 0.89)	
Overall population	85 (7.58)	156 (15.54)	-7.96 (-8.85 to -7.08)	0.71 (0.55 to 0.93)	
**GLP-1RA vs DPP-4i**					
**MACE**					
With NAFLD	26 (10.93)	114 (28.35)	-17.4 (-22.2 to -12.6)	0.51 (0.32 to 0.82)	0.9452
Without NAFLD	65 (21.04)	258 (34.81)	-13.8 (-18.3 to -9.23)	0.52 (0.39 to 0.70)	
Overall population	91 (17.30)	372 (32.53)	-15.2 (-18.6 to -11.9)	0.52 (0.40 to 0.66)	
**HHF**					
With NAFLD	10 (6.01)	25 (24.06)	-18.1 (-22.0 to -14.1)	0.94 (0.42 to 2.07)	0.3978
Without NAFLD	11 (16.50)	46 (28.03)	-11.5 (-15.6 to -7.50)	0.60 (0.31 to 1.18)	
Overall population	21 (12.62)	71 (26.63)	-14.0 (-16.9 to -11.1)	0.73 (0.44 to 1.21)	

Abbreviation: CI, confidence interval; DPP-4i, dipeptidyl peptidase 4 inhibitors; GLP-1RA, glucagon-like peptide-1 receptor agonists; HHF, hospitalization for heart failure; MACE, major adverse cardiovascular events; NAFLD, non-alcoholic fatty liver disease; SGLT-2i, sodium glucose cotransporter 2 inhibitors

© 2023 Bea S et al. *JAMA Network Open.*

eAppendix 1. Definitions of Proxy Indicator for NAFLD Definition[*]

1. Main analysis

Fatty liver index (FLI) is a non-invasive and simple method to obtain and may help clinicians to screen patients with non-alcoholic fatty liver disease. FLI ≥60 can be used to estimate presence of NAFLD with specificity of 86%[*].

FLI was calculated using formula as follow:

$$FLI = (e^{0.953*loge\ (triglycerides) + 0.139*BMI + 0.718*loge\ (ggt) + 0.053*waist\ circumference - 15.745}) / (1 + e^{0.953*loge\ (triglycerides) + 0.139*BMI + 0.718*loge\ (ggt) + 0.053*waist\ circumference - 15.745}) * 100$$

According to previous validations, FLI score below 30 excludes the presence of NAFLD (ruled out), while an FLI score equal to or greater than 60 confirms the presence of NAFLD (ruled in). However, NAFLD is a disease with a continuous spectrum, so the several studies defined mild NAFLD as 30≤FLI<60 and severe NAFLD as 60≤FLI. To follow previous validation studies and to ensure a robust definition of NAFLD patients, we used 60≤FLI as the definition of NAFLD patients.

2. Sensitivity analysis

The Hepatic Steatosis Index (HSI) represents an effective screening tool for identifying individuals with NAFLD. Its application can aid in the selection of individuals who would benefit from liver ultrasonography, as well as guide decisions regarding the implementation of lifestyle modifications to manage the condition. HSI ≥36 can be used as proxy indicator for presence of NAFLD with PPV of 85.9%[†].

HSI was calculated using formula as follow:

HSI=8*ALT/AST ratio + BMI + (+2, if DM; +2 if female)

[Reference]

[*]Bedogni G, Bellentani S, Miglioli L, Masutti F, Passalacqua M, Castiglione A, Tiribelli C. The Fatty Liver Index: a simple and accurate predictor of hepatic steatosis in the general population. BMC Gastroenterol. 2006 Nov 2;6:33. doi: 10.1186/1471-230X-6-33.

© 2023 Bea S et al. *JAMA Network Open.*

[†]Lee JH, Kim D, Kim HJ, Lee CH, Yang JI, Kim W, Kim YJ, Yoon JH, Cho SH, Sung MW, Lee HS. Hepatic steatosis index: a simple screening tool reflecting nonalcoholic fatty liver disease. Dig Liver Dis. 2010 Jul;42(7):503-8. doi: 10.1016/j.dld.2009.08.002. Epub 2009 Sep 18.

eAppendix 2. Description of Sensitivity Analyses

Discussions

The findings across a range of sensitivity analyses were generally consistent with those of the main analysis in both of SGLT-2i and GLP-1RA cohorts. In particular, we observed the similar results in another model estimating NAFLD, the hepatic steatosis index (HSI), which takes into account sex and type 2 diabetes prevalence. Thus, it is more likely used as an indicator of the robustness of our findings in assessing the CVD effectiveness of glucose lowering medications among patients with type 2 diabetes according to presence or absence of NAFLD. Moreover, similar results were observed in the analysis of restricted cohort within one year of fatty liver index. These results suggested that the duration between the measurement date of the health examination record for NAFLD estimation and cohort entry had a limited impact on the results.

© 2023 Bea S et al. *JAMA Network Open.*

eAppendix 3. Description of Exploratory Analyses*

This study evaluated the safety profiles of two novel antidiabetic drugs of SGLT-2i and GLP-1RA among patients with NAFLD. This study's association between SGLT-2i and genital infection was in line with previous research (incidence rate ratio 3.50, 95% CI 3.09-3.95) *. With regards to DKA, our results showed higher estimates of DKA among patients with NAFLD, indicating that abnormal hepatic glucose metabolism may further increase the risk of DKA with SGLT-2i in this subgroup, which are corroborated by a meta-analysis of seven large-scale randomized trials (RR 3.54, 95% CI 0.82-15.39)[†]. Meanwhile, GLP-1RA was associated with a decrease risk of hypoglycemia in the overall population, and the point estimate for hypoglycemia in patients with NAFLD were lower than those of without NAFLD. This effect could be attributed to the reported improvement of NAFLD by GLP-1RA in other studies, which may lead to an improvement in the blood glucose control function of the liver[‡]. Taken together, the overall safety profiles of both SGLT-2i and GLP-1RA among patients with NAFLD were favorable. However, further research is warranted to establish conclusive evidence on the association between these novel antidiabetic drugs and the risk of adverse events in this subgroup.

[References]

*Marilly E, Cottin J, Cabrera N, Cornu C, Boussageon R, Moulin P, et al. SGLT2 inhibitors in type 2 diabetes: a systematic review and meta-analysis of cardiovascular outcome trials balancing their risks and benefits. Diabetologia. 2022;65(12):2000-10.

[†]Kaze AD, Zhuo M, Kim SC, Patorno E, Paik JM. Association of SGLT2 inhibitors with cardiovascular, kidney, and safety outcomes among patients with diabetic kidney disease: a meta-analysis. Cardiovasc Diabetol. 2022;21(1):47.

[‡]Newsome PN, Buchholtz K, Cusi K, Linder M, Okanoue T, Ratziu V, et al. A Placebo-Controlled Trial of Subcutaneous Semaglutide in Nonalcoholic Steatohepatitis. N Engl J Med. 2021;384(12):1113-24.

© 2023 Bea S et al. *JAMA Network Open.*

eFigure 1. Flowchart of Study Population Selection Among New Users of SGLT-2 Inhibitors vs DPP-4 Inhibitors With Varying NAFLD Status

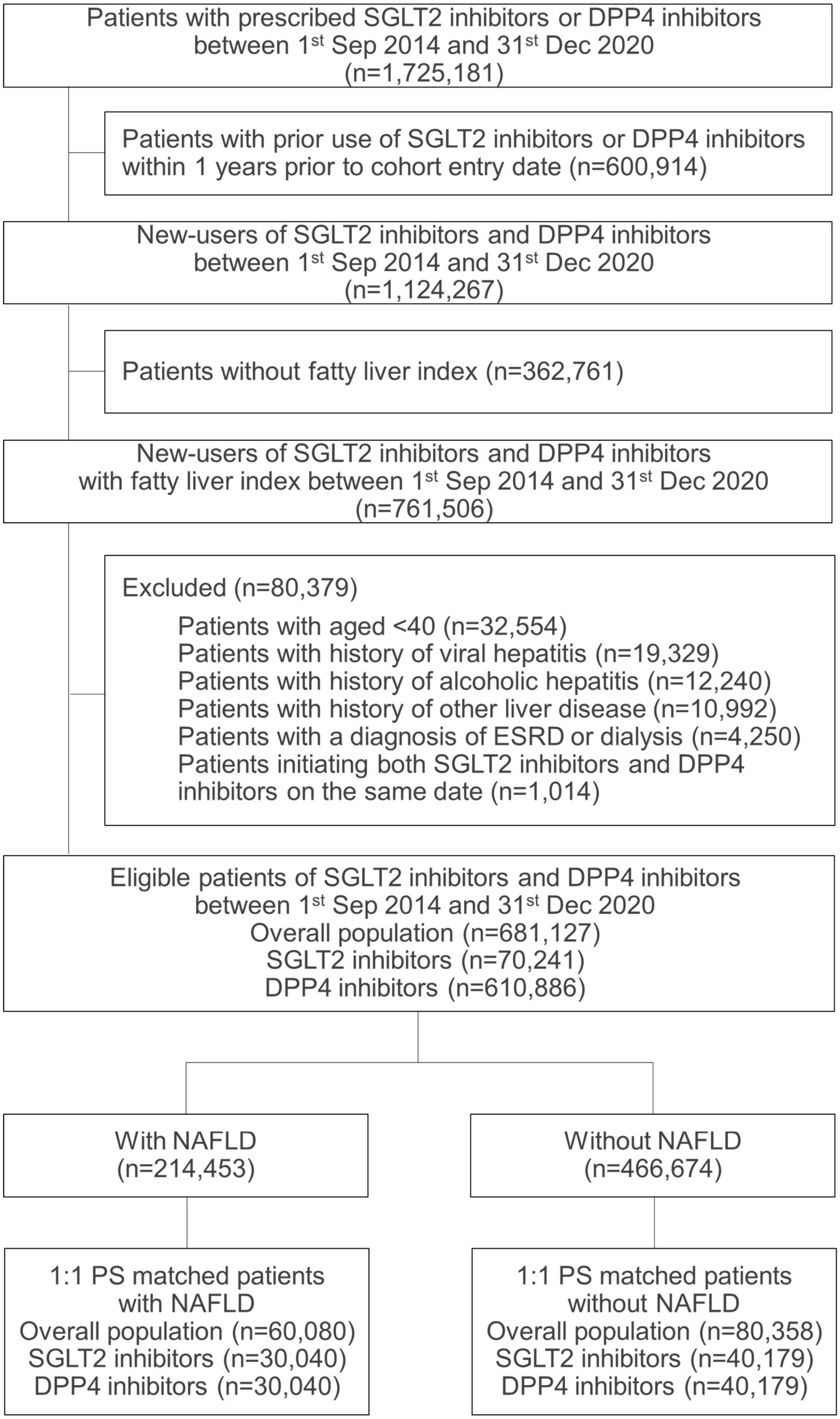

© 2023 Bea S et al. *JAMA Network Open.*

eFigure 2. Flowchart of Study Population Selection Among New Users of GLP-1RAs vs DPP-4 Inhibitors With Varying NAFLD Status

© 2023 Bea S et al. *JAMA Network Open.*

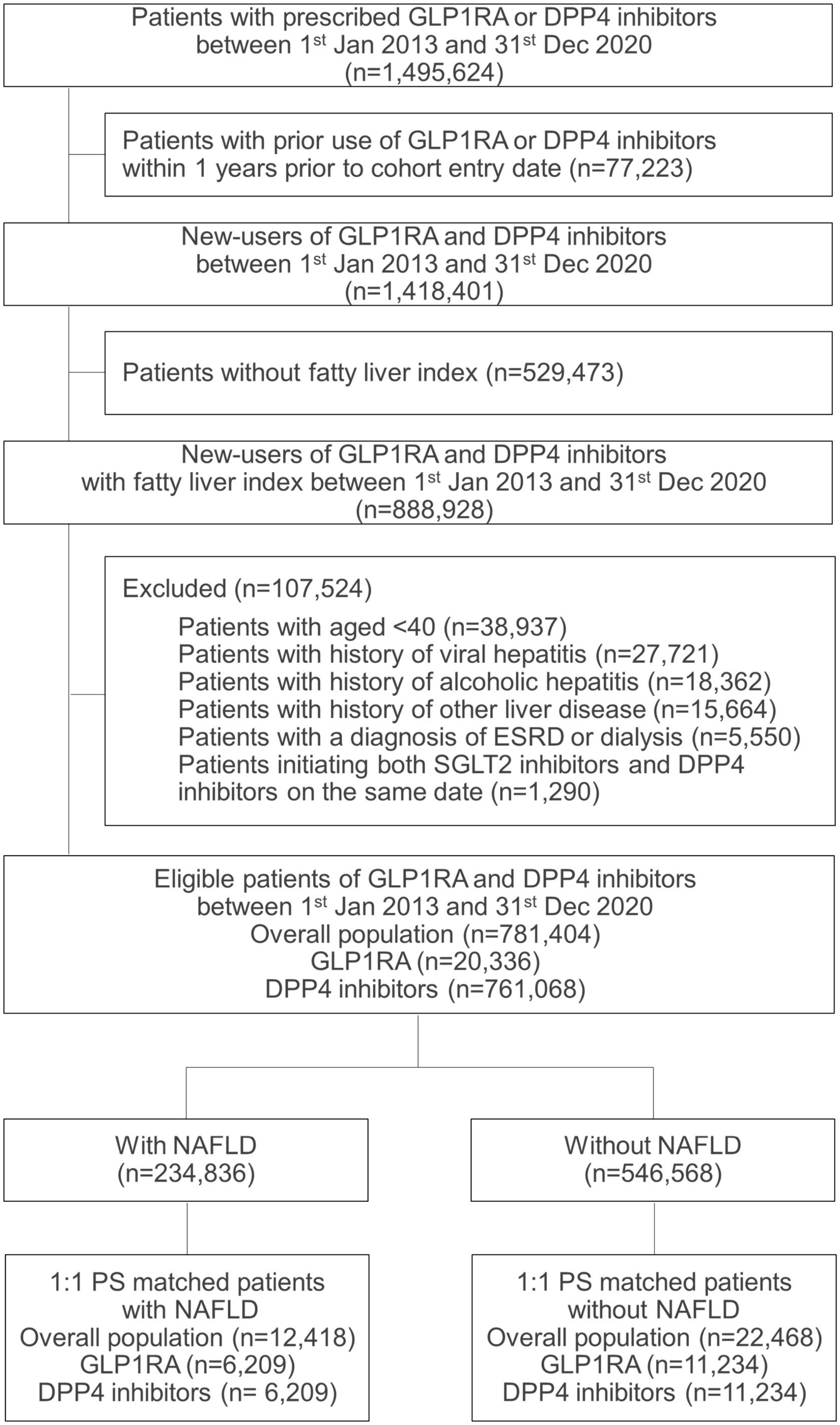

© 2023 Bea S et al. *JAMA Network Open.*

eFigure 3. Results of Exploratory Analyses*

	Events, n (Incidence Rate per 1000 Person-Years)		Rate Difference per 1000 Person-Years (95% CI)	Hazard Ratio (95% CI)	Forest Plot	P value for Homogeneity
	Drugs of Interest	DPP-4 inhibitor				
**SGLT-2i versus DPP-4i**						
**Amputation**						
With NAFLD	4 (0.09)	15 (0.31)	-0.22 (-0.40 to -0.04)	0.29 (0.09 to 0.86)		0.4513
Without NAFLD	12 (0.21)	29 (0.40)	-0.19 (-0.38 to -0.01)	0.48 (0.25 to 0.95)		
Overall population	16 (0.16)	44 (0.37)	-0.21 (-0.34 to -0.08)	0.41 (0.23 to 0.73)		
**Genital infection**						
With NAFLD	2702 (67.39)	1483 (32.36)	35.03 (32.00 to 38.06)	1.98 (1.86 to 2.11)		0.7018
Without NAFLD	5208 (104.3)	3229 (48.43)	55.89 (52.60 to 59.18)	1.95 (1.86 to 2.04)		
Overall population	7910 (87.87)	4712 (41.88)	45.99 (43.71 to 48.26)	1.94 (1.87 to 2.01)		
**AKI**						
With NAFLD	69 (1.57)	179 (3.73)	-2.17 (-2.83 to -1.51)	0.41 (0.31 to 0.54)		0.1286
Without NAFLD	111 (1.93)	243 (3.37)	-1.45 (-2.00 to -0.89)	0.54 (0.43 to 0.67)		
Overall population	180 (1.77)	422 (3.52)	-1.75 (-2.17 to -1.32)	0.48 (0.41 to 0.58)		
**Non-vertebral fracture**						
With NAFLD	1124 (26.37)	1237 (26.69)	-0.31 (-2.46 to 1.83)	0.98 (0.90 to 1.06)		0.2592
Without NAFLD	1612 (29.02)	2100 (30.45)	-1.43 (-3.35 to 0.50)	0.91 (0.86 to 0.97)		
Overall population	2736 (27.87)	3337 (28.94)	-1.07 (-2.50 to 0.37)	0.93 (0.89 to 0.98)		
**DKA**						
With NAFLD	7 (0.16)	2 (0.04)	0.12 (-0.01 to 0.25)	3.69 (0.77 to 17.79)		0.1367
Without NAFLD	18 (0.31)	21 (0.29)	0.02 (-0.17 to 0.21)	1.02 (0.54 to 1.92)		
Overall population	25 (0.25)	23 (0.19)	0.05 (-0.07 to 0.18)	1.24 (0.70 to 2.19)		
**GLP-1RA versus DPP-4i**						
**GI disorder**						
With NAFLD	32 (8.77)	72 (7.48)	1.29 (-2.20 to 4.79)	0.96 (0.62 to 1.49)		0.5417
Without NAFLD	50 (8.04)	155 (8.80)	-0.75 (-3.38 to 1.87)	0.81 (0.59 to 1.13)		
Overall population	82 (8.31)	227 (8.33)	-0.02 (-2.12 to 2.08)	0.86 (0.66 to 1.12)		
**Pancreatitis**						
With NAFLD	58 (15.95)	90 (9.37)	6.58 (2.04 to 11.12)	1.20 (0.85 to 1.70)		0.4431
Without NAFLD	119 (19.25)	226 (12.90)	6.35 (2.51 to 10.20)	1.02 (0.81 to 1.28)		
Overall population	177 (18.03)	316 (11.65)	6.38 (3.43 to 9.33)	1.07 (0.88 to 1.29)		
**Hypoglycemia**						
With NAFLD	7 (1.91)	36 (3.73)	-1.82 (-3.68 to 0.05)	0.45 (0.20 to 1.04)		0.2973
Without NAFLD	37 (5.94)	111 (6.28)	-0.34 (-2.58 to 1.90)	0.73 (0.50 to 1.08)		
Overall population	44 (4.45)	147 (5.38)	-0.93 (-2.51 to 0.64)	0.66 (0.46 to 0.94)		

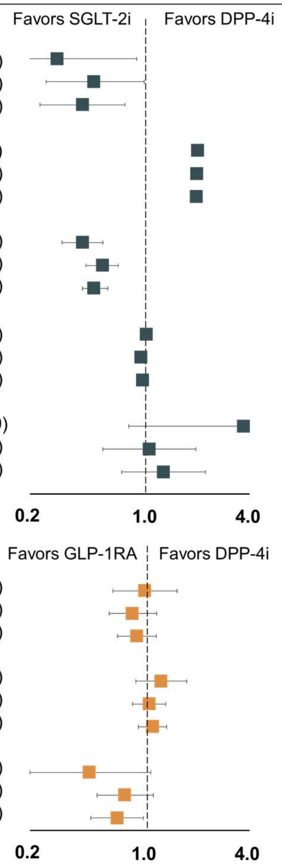

Abbreviation: AKI, acute kidney injury; CI, confidence interval; DKA, diabetic ketoacidosis; DPP-4i, dipeptidyl peptidase 4 inhibitors; GI, gastrointestinal; GLP-1RA, glucagon-like peptide-1 receptor agonists; NAFLD, non-alcoholic fatty liver disease; SGLT-2i, sodium glucose cotransporter 2 inhibitors

© 2023 Bea S et al. *JAMA Network Open.*
