# Supplementary material for: Outcomes of SGLT-2i and GLP-1RA Therapy Among Patients With Type 2 Diabetes and Varying NAFLD Status
Source: JAMA Netw Open. 2023 Dec 28;6(12):e2349856. doi: 10.1001/jamanetworkopen.2023.49856 (PMC10755620; doi:10.1001/jamanetworkopen.2023.49856)
Supplement: Supplement 2. — Data Sharing Statement [file jamanetwopen-e2349856-s002.pdf]

Data Sharing Statement

Bea. Outcomes of SGLT-2i and GLP-1RA Therapy Among Patients With Type 2 Diabetes and Varying NAFLD Status. *JAMA Netw Open*. Published January 02, 2024. doi:10.1001/jamanetworkopen.2023.49856

Data
Data available: No
